# Transcriptomic Analyses of *Scrippsiella trochoidea* Reveals Processes Regulating Encystment and Dormancy in the Life Cycle of a Dinoflagellate, with a Particular Attention to the Role of Abscisic Acid

**DOI:** 10.3389/fmicb.2017.02450

**Published:** 2017-12-11

**Authors:** Yunyan Deng, Zhangxi Hu, Lixia Shang, Quancai Peng, Ying Zhong Tang

**Affiliations:** ^1^CAS Key Laboratory of Marine Ecology and Environmental Sciences, Institute of Oceanology, Chinese Academy of Sciences, Qingdao, China; ^2^Laboratory of Marine Ecology and Environmental Science, Qingdao National Laboratory for Marine Science and Technology, Qingdao, China; ^3^Research Center of Analysis and Measurement, Institute of Oceanology, Chinese Academy of Sciences, Qingdao, China

**Keywords:** resting cyst, harmful algal blooms (HABs), dormancy, UHPLC-MS/MS, abscisic acid (ABA), dinoflagellate, *Scrippsiella trochoidea*

## Abstract

Due to the vital importance of resting cysts in the biology and ecology of many dinoflagellates, a transcriptomic investigation on *Scrippsiella trochoidea* was conducted with the aim to reveal the molecular processes and relevant functional genes regulating encystment and dormancy in dinoflagellates. We identified via RNA-seq 3,874 (out of 166,575) differentially expressed genes (DEGs) between resting cysts and vegetative cells; a pause of photosynthesis (confirmed via direct measurement of photosynthetic efficiency); an active catabolism including β-oxidation, glycolysis, glyoxylate pathway, and TCA in resting cysts (tested via measurements of respiration rate); 12 DEGs encoding meiotic recombination proteins and members of MEI2-like family potentially involved in sexual reproduction and encystment; elevated expressions in genes encoding enzymes responding to pathogens (chitin deacetylase) and ROS stress in cysts; and 134 unigenes specifically expressed in cysts. We paid particular attention to genes pertaining to phytohormone signaling and identified 4 key genes regulating abscisic acid (ABA) biosynthesis and catabolism, with further characterization based on their full-length cDNA obtained via RACE-PCR. The qPCR results demonstrated elevated biosynthesis and repressed catabolism of ABA during the courses of encystment and cyst dormancy, which was significantly enhanced by lower temperature (4 ± 1°C) and darkness. Direct measurements of ABA using UHPLC-MS/MS and ELISA in vegetative cells and cysts both fully supported qPCR results. These results collectively suggest a vital role of ABA in regulating encystment and maintenance of dormancy, akin to its function in seed dormancy of higher plants. Our results provided a critical advancement in understanding molecular processes in resting cysts of dinoflagellates.

## Introduction

Harmful algal blooms (HABs) have been receiving mounting attention worldwide due to their global increase in frequency, duration, and distribution during the last decades (Anderson et al., 2012). Dinoflagellates are a group of unicellular eukaryotes notorious for being the most common causative agents of HABs (Taylor et al., 2008). Nearly 200 (~10% of the total) dinoflagellate species cause HABs, which account for 75% of all harmful phytoplankton species (Smayda, 1997).

Dinoflagellates display an amazing range of ecological adaptation through multiple adaptive strategies (Taylor et al., 2008). Several hypotheses have been put forward to account for their ubiquitous distribution and recurrent blooms, such as mixotrophy (Stoecker, 1999), allelopathic effect (Legrand et al., 2003), toxin production (Turner and Tester, 1997), and stimulation by vitamins (Tang et al., 2010). The formation of resting cysts is one of the survival strategies for some dinoflagellate species. The resting cysts of dinoflagellates are the benthic, dormant stage usually resulted from sexual reproduction (Dale, 1983), the formation of which is accompanied by a series of cellular processes such as gamete formation, sexual mating, cell fusion, and cell morphogenesis (Bravo and Figueroa, 2014; Tang et al., 2016). Resting cysts play pivotal roles in the biology and ecology of dinoflagellates, particularly the processes of HABs, as they are associated with genetic recombination, bloom initiation, bloom termination, bloom recurrence, and geographic expansion (Dale, 1983; Anderson et al., 1984; Smayda, 2007; Bravo and Figueroa, 2014; Tang and Gobler, 2015; Tang et al., 2016). Resting cysts in sediments can resist harsh environmental conditions and are protected from viruses, grazers and parasite attacks (Ribeiro et al., 2011; Bravo and Figueroa, 2014). Encystment is usually considered to be an adaptive response to environmental stresses, such as nutrient depletion (Anderson et al., 1984; Grigorszky et al., 2006), changes in temperature (Sgrosso et al., 2001; Grigorszky et al., 2006), salinity (Zonneveld and Susek, 2007), day length (Sgrosso et al., 2001), high cell densities (Garcés et al., 2004) and bacterial attack (Lundgren and Granéli, 2011). However, little has been understood about the endogenous regulatory factors and physiological mechanisms for resting cyst fosrmation and germination (i.e., encystment and excystment) (Tang et al., 2016). More specifically, the genes and their expressions underlying the biological processes have not yet been investigated.

Dinoflagellates are the second largest group of eukaryotic photoautotrophs, yet there has only been a slow accumulation of genomic information of this diverse group, mainly because of their extremely large nuclear genomes (up to 250 Gb), high methylated nucleotides, and unusual regulatory mechanisms, features implying that the gene-regulatory systems of dinoflagellate might be very different from other eukaryotes (Lin, 2011; Murray et al., 2016). These peculiar genetic features make the global genomic sequencing highly challenging. Consequently, only 3 partial genomes of dinoflagellate species exist, all corresponding to members of the genus *Symbiodinium* (Shoguchi et al., 2013; Lin et al., 2015; Aranda et al., 2016). Compared to the whole-genome sequencing, characterization of transcriptome is an efficient approach to understand functions of a genome at particular physiological states. Previous transcriptomic works on dinoflagellates were mainly focused on species of *Alexandrium, Karenia*, and *Symbiodinium* and on the mechanisms pertaining to toxin synthesis, responses to a variety of environmental conditions or nutrients, and symbiosis (Van Dolah et al., 2007; Moustafa et al., 2010; Wisecaver and Hackett, 2010; Yang et al., 2010; Jaeckisch et al., 2011; Leggat et al., 2011; Lowe et al., 2011; Morey et al., 2011; Bayer et al., 2012; Roy et al., 2014; Zhang et al., 2014; Xiang et al., 2015; Cooper et al., 2016; Guo et al., 2016; Levin et al., 2016; Parkinson et al., 2016). RNA-Seq (high-throughput RNA-sequencing) is a revolutionary tool using deep-sequencing technologies for the transcriptome profiling, with advantages of ultra-high throughput, accurate measurement, high sensitivity, and large dynamic range (Wang et al., 2009). Combined with *de novo* transcripts assembly, the method is particularly attractive for non-model organisms without reference genomic information and has recently been applied to transcriptomic studies on dinoflagellate species of 6 genera (Roy et al., 2014; Zhang et al., 2014; Xiang et al., 2015; Cooper et al., 2016; Guo et al., 2016; Levin et al., 2016; Parkinson et al., 2016).

The seed dormancy of higher plants is a temporary block of germination even under favorable physical conditions (Koornneef et al., 2002), which is similar to the mandatory dormancy of resting cyst of dinoflagellates. Seed dormancy and germination are controlled by a large number of genes, which are affected by both developmental and environmental cues (Kucera et al., 2005). Phytohormones are a class of chemical molecules that are produced in extremely low concentrations and serve as signal messengers to coordinate cellular activities (Lu and Xu, 2015). They are extremely important for the regulation of seed dormancy and germination (Brady and McCourt, 2003; Kucera et al., 2005; Footitt et al., 2011). Abscisic acid (ABA) is such a hormone that plays a prominent role in controlling seed dormancy and germination. In many land plants, endogenous ABA is involved in the initiation and maintenance of seed dormancy (Cutler and Krochko, 1999; Koornneef et al., 2002; Brady and McCourt, 2003; Kucera et al., 2005; Nambara and Marion-Poll, 2005; Footitt et al., 2011; Kiseleva et al., 2012). In algae, although ABA has been found in a broad spectrum of algal lineages (as reviewed in Lu and Xu, 2015), studies on ABA-mediated dormancy or cyst formation in algae are rare. Only Kobayashi et al. (1997a) reported that exogenous ABA induced morphological change from green vegetative cells to “red mature cyst cells” of the unicellular green alga *Haematococcus pluvialis* and it was therefore speculated that ABA might regulate the transformation between two stages. However, it is worth pointing out that the “red mature cyst cells” were formed via asexual reproduction (Kobayashi et al., 1997b) and are similar to “akinetes” in cyanophytes (Herdman, 1988), rather than the resting cysts of dinoflagellate primarily resulted from sexual reproduction.

Endogenous ABA concentrations are the result of a dynamic balance between continuous synthesis and catabolism (Cutler and Krochko, 1999), and, therefore, the active hormone level that control physiological processes results from either changes in synthesis or in catabolism (Nambara and Marion-Poll, 2005). Regarding ABA biosynthesis in higher plants, the “indirect pathway” is the primary pathway (Schwartz et al., 2003), in which zeaxanthin epoxidase (ZEP) is regarded as a crucial enzyme for catalyzing the first step of reactions (Marin et al., 1996). Antisense and sense *ZEP* transformation of *Nicotiana plumbaginifolia* lead to decreased and increased ABA biosynthesis and seed dormancy, respectively (Frey et al., 1999). The cleavage of 9-*cis*-epoxycarotenoids to xanthoxin, catalyzed by 9-*cis*-epoxycarotenoid dioxygenases (NCED), is proposed to be the vital speed-determining step of the pathway (Schwartz et al., 1997). The *nced6*/*nced9* double-mutants seed of *Arabidopsis* showed declined ABA level accompanied with reduced dormancy (Lefebvre et al., 2006), while over-expression of *NCED* in tomato caused ABA accumulation and enhanced seed dormancy (Thompson et al., 2000). The abscisic-aldehyde oxidase (AAO), responsible for the final step, is a target of self-regulatory loop regulating ABA biosynthesis (Xiong et al., 2001). The *aao3* mutants of *Arabidopsis* with declined seed ABA biosynthesis presented reduced dormancy (Gonzalez-Guzman et al., 2004). On the other hand, hydroxylation at C-8′ position is the predominant pathway of ABA catabolism in terrestrial plants (Kushiro et al., 2004; Kucera et al., 2005). The ABA-8′-hydroxylase (ABAH), encoded by members of CYP707A family, accounts for the committed step and plays regulatory role in the control of ABA content (Saito et al., 2004). Enhanced dormancy was evident in *Arabidopsis cyp707a2* mutants with increased ABA content due to the block of ABA catabolism in seed (Kushiro et al., 2004). For algal lineages, information about ABA synthesis and catabolism is rather fragmentary. Although evidences suggest that the ABA biosynthetic and metabolic pathways in algae resemble those in higher plants (Kiseleva et al., 2012; Lu and Xu, 2015), gaps remained in our knowledge about the sequences of homologous genes in dinoflagellates, not to mention their molecular characteristics and physiological functions.

The thecate dinoflagellate *Scrippsiella trochoidea* (Stein) Loeblich III 1976 was adopted in this study as a model HAB species and resting cyst producer, because: it is a cosmopolitan microalga (Steidinger and Tangen, 1997) that commonly forms dense blooms worldwide (as reviewed in Tang and Gobler, 2012) and has been shown to cause rapid lethal effects on shellfish (*Crassostrea virginica* and *Mercenaria mercenaria*) larvae (Tang and Gobler, 2012); More importantly, *S. trochoidea* is well-known for being able to easily produce resting cysts both in laboratory and field (Qi et al., 1997) and having abundant cysts in marine sediment bed. Also, it is noteworthy that, in comparing with those cysts having a cellulosic wall, the calcareous wall of *S. trochoidea* cyst is more resistant to harsh environmental condition in sediment (Janofske, 2000; Shin et al., 2013) and that is possibly why cysts of *S. trochoidea* represent the most dominant species in sediment beds of coastal sediments (Qi et al., 1997; Wang et al., 2007; Satta et al., 2010); and the length of mandatory dormancy for *S. trochoidea* (~15–60 days; Binder and Anderson, 1987; Kim and Han, 2000) is relatively short and thus manageable for our laboratory work. Therefore, although the species exhibited tremendous genetic diversity (Montresor et al., 2003; Zinssmeister et al., 2011), *S. trochoidea* is still a good model organism for the study of molecular mechanisms regulating the life cycle transitions of dinoflagellates.

In this study, we used *S. trochoidea* as the model organism to investigate its transcriptomes in resting cysts and vegetative cells with the goal of finding functional genes pertaining to regulating the alteration of life cycles in dinoflagellates by applying RNA-Seq technology. After obtaining the full-length cDNA sequences of 4 crucial genes involved in ABA biosynthesis and catabolism via RACE (rapid-amplification of cDNA ends), their expressions in vegetative cells and resting cysts at different conditions were quantified by qPCR with the aims to correlate the gene expressions to endogenous ABA levels, which were also measured with UHPLC-MS/MS (ultra-high performance liquid chromatography-tandem mass-spectrometry) and ELISA (enzyme linked immunosorbent assay) assays. Our results provided important insights into the gene expression landscape for resting cysts of dinoflagellate and paved the way for future identification of specific functional genes contributing to the processes of encystment and excystment.

## Materials and methods

### Algal culture and samples preparation

The culture of *S. trochoidea* (strain IOCAS-St-1) was obtained from the Marine Biological Culture Collection Centre, Institute of Oceanology, Chinese Academy of Sciences, which was isolated from the Yellow Sea of China and was identified using partial 18S and 28S rDNA sequence (see Data S7). Cultures were routinely maintained in f/2 (-Si) medium (Guillard, 1975) supplemented with 10^−8^ M (final concentration) selenium and PII metal mix of GSe medium (Doblin et al., 1999), made with sterile 0.22 μm-filtered seawater (salinity 32–33), at 20 ± 1°C in an incubator with a 12:12 h L/D cycle and a light intensity of 100 μmol photons m^−2^ s^−1^. A penicillin-streptomycin mix (100×, Solarbio, Beijng, China) was added into the medium immediately before inoculation (final concentration 3%) to discourage bacterial growth.

Cultures at exponential phase were inoculated into 500 mL flasks containing 300 mL medium to achieve an initial cell density of ~1 × 10^3^ cells mL^−1^ and then grown under the culturing conditions as routine maintenance (*n* = 3). The 15 days-old cultures were transferred into 6-well culture plates (Corning, US; 10 mL in each well) for resting cysts production and checked every 2 days under an Olympus IX73 inverted microscope. Immature cysts and mature resting cysts were identified according to Qi et al. (1997) and Tang and Gobler (2012). For cDNA library preparation, measurements of photosynthesis and respiration rate, and endogenous ABA quantification, mature resting cysts were obtained from the cultures that had been inoculated for about 50–60 days. They were washed with sterile filtered seawater several times until no vegetative cell and planozygotes observed in the samples by checking under microscope. The long duration of culturing and the washing steps made the majority of cysts (>95%) were mature cysts as observed under a light microscope before RNA extraction. For qPCR analyses, immature cysts and mature cysts were isolated by micro-pipetting under light microscope (see Methods S1, S2).

### Preparation of cDNA library for RNA-seq

For each sample of vegetative cells and resting cysts (labeled as Vegetative cell-a, -b, -c, Cyst-a, -b, -c), about 400 mg (wet weight) fresh biomass was harvested from more than 20 flasks each containing 300 mL culture with a cell density of ~6–7 × 10^3^ cells mL^−1^ for total RNA extraction using RNeasy Plant Mini Kit (QIAGEN, Germany), and was treated with RNase-Free DNase Set (QIAGEN, Germany) to remove residual genomic DNA. The quantity and quality of total RNA were analyzed with 1% agarose gel electrophoresis and NanoDrop™ 1,000 spectrophotometer (Thermo Fisher Scientific, USA). RNA integrity of all samples was confirmed using Agilent Technologies 2,100 Bioanalyzer (Agilent Technologies, CA, USA), and only those extracts with a minimum RIN value ≥ 6 were used for the subsequent processes. Poly-(A)-containing mRNA were purified from total RNA using oligo-(dT) conjugated magnetic beads (Illumina, USA). The cDNA libraries were generated with the captured mRNA using Illumina TruSeq RNA Sample Preparation Kit (Illumina, San Diego, USA) following manufacturer's guidelines and then sequenced via the Illumina HiSeq™ 2,000 platform that generated about 90 bp paired-end raw reads (see Methods S1).

### Transcriptome data processing, *de Novo* assembly

Raw data of Illumina sequencing were obtained after base calling and stored in fastq format. Cleaning steps of the raw reads were as follows: (1) trimming adapter sequences. (2) removing the reads containing unknown nucleotides (Ns) >5. (3) filtering the low-quality reads with quality value ≤10 is more than 20%. All subsequent analyses were based on the remaining clean reads.

Due to the absence of genomic information, *de novo* assembly was performed with the Trinity software in referring to the strategy of Grabherr et al. (2011). The clean reads were firstly assembled into contigs and then transcripts with Trinity which recovers more full-length transcripts across a broad range of expression levels, with sensitivity similar to methods that rely on genome alignments compared with other *de novo* transcriptome assemblers (Grabherr et al., 2011). Based on the overlap between the reads to produce longer contigs, the contigs were then joined into transcripts. If a component had more than one transcript, the longest one was selected to represent assembled transcripts (i.e., unigenes). The six samples were first assembled separately, and the transcripts of the six samples were mixed together for clustering to make a database including all unigenes. The program TGICL was used to acquire a single set of non-redundant unigenes (Pertea et al., 2003).

### Identification of DEGs between resting cysts vs. vegetative cells

Raw sequencing data were processed, *de novo* assembled, and annotated as described in Methods S1. Then the abundance of unigenes was normalized and calculated using uniquely mapped reads by the RPKM (reads per kilobase per million reads) method (Mortazavi et al., 2008). *P*-value and FDR (false discovery rate) were used to determine differentially expressed genes (DEGs) (Benjamini et al., 2001). Values of *p* ≤ 0.05, FDR ≤ 0.001, and log_2_Ratio ≥ 1 were set as the thresholds for DEGs. Differential expression analysis between resting cysts vs. vegetative cells was performed by modeling count data with negative binomial distributions described in the DESeqmethod (Anders and Huber, 2010). For pathway enrichment analysis, all DEGs were mapped to categories in Kyoto Encyclopedia of Genes and Genomes database (KEGG) database and searched for significantly enriched metabolic pathways or signal transduction pathways in DEGs comparing with the whole transcriptome background. The *p*-values were calculated using hypergeometric test and went through multiple testing corrections (Kanehisa et al., 2007). A threshold of corrected *p* ≤ 0.05 was applied to call KEGG categories as “significantly enriched.”

### Cloning of genes involved in ABA biosynthesis and catabolism

The full-length cDNA sequences of 4 genes involved in ABA biosynthesis and catabolism, *NCED, ZEP, ABAH*, and *AAO*, from *S. trochoidea* (designated as *StNCED, StZEP, StABAH*, and *StAAO*, respectively) were isolated based on the transcriptome data and using RACE-PCR. For fragments amplification, PCR protocol followed that described in Deng et al. (2015) with specific primers shown in Table S12. For RACE-PCR, Smarter^TM^ RACE cDNA Amplification Kit (Clontech, USA) was used according to the manufacturer's standard protocol. Nested-PCR amplifications were conducted with specific primers (Table S12). After reactions, all products were run on 1% agarose gel. The bands of interest were purified by agarose gel DNA fragment recovery kit (TaKaRa, Tokyo, Japan), ligated with p*EASY*-T1 cloning vector (TransGen Biotech, Beijing, China), and then sequenced (BGI, Beijing, China).

### Transcriptional profiles of genes involved in ABA biosynthesis and catabolism with qPCR detection

For qPCR analyses of genes involved in ABA biosynthesis and catabolism, 3 arrays of experiments were conducted: the first one was for cells that were harvested at different life stages, including vegetative cells, immature cysts, mature resting cysts, and resting cysts maintained for 1 month (mo.); the second one was for mature resting cysts that were kept at conditions the same as culture maintenance in the original plates for 0, 1, 2, 3, 4, 5, and 6 mo.; and the third one was for mature resting cysts that were kept at 4 ± 1°C in darkness for 0, 1, 2, 3, 4, 5, and 6 mo., respectively (see Methods S1). All samples were prepared in triplicates. Based upon pre-screening for genes with expression stability (Data S3), 3 reference genes (*MDH, LBP*, and *UBC*) were used in the subsequent qPCR analyses (Methods S1). The relative expression levels were analyzed using the 2^−ΔΔCt^ method (Schmittgen et al., 2000) and expressed as a dimensionless unit normalized with the expressions of reference genes, respectively. Data were presented in graphs as mean ± standard deviation (SD), subjected to one-way analysis of variance (ANOVA) and a subsequent Tukey's honestly significant difference test. Significance was accepted when *p* ≤ 0.05.

### qPCR verification of selected RNA-seq unigenes

A total of 12 unigenes of interest identified by RNA-seq, including catalase (*CAT*), cold shock protein (*CSP*), mitogen-activated protein kinase 6 (*MPK6*), gibberellin receptor (*GID1*), Cyclin B, cytochrome b6 (*petB*), ribulose-1, 5-bisphosphate carboxylase/oxygenase (*Rubisco*), fructose-1,6-bisphosphatase (*FBP*), histidine kinase (*HK*), pyruvate carboxylase (*PC*), transcriptional regulator (*TR*), and a unigene specifically expressed in resting cysts but without annotation (Unigene 53202), were selected for experimental verification for their differential expression (Table S12). Biological triplicate reactions together with non-template reaction (NTC) of each gene were performed on a single plate. To confirm correct amplification, the qPCR products were migrated on 1% agarose gel and sequenced. The relative expression level calculations and statistical analyses were conducted referring to Deng et al. (2016).

### Photosynthesis and respiration rate measurements

Photosynthesis and respiration rates of resting cysts and vegetative cells were quantified as follows: The apparent PSII photochemical quantum efficiency (*F*v/*F*m) defined by Kolber et al. (1998) was measured using a FastOcean plus Act2 Fast Repetition Rate fluorometer (Chelsea Technologies Group Ltd, UK). The fluorescence measurement protocol consisted of 3 consecutive acquisitions, with each acquisition consisting of 60 repetitions of the following sequence: 100 flashlets at 1-μs intervals to reach saturation, and 50 flashlets at 49-μs intervals to record relaxation kinetics. All curve fits and fluorescence transients were manually inspected in real time. For measurements of respiration rate, measurements of oxygen consumption were performed in a Liquid-Phase Oxygen Electrode Chamber (Hansatech Instruments, Norfolk, England). Briefly, the solid bicarbonate was added to the electrode chamber to ensure the consumption of all oxygen and determine the baseline. About 2 mL suspension of vegetative cells or cysts (~1 × 10^5^ cells mL^−1^) was added to the chamber. Respiration rates were then calculated from the slope of regression of oxygen concentration against time.

### Quantification of endogenous ABA in vegetative cells and resting cysts using ELISA and UHPLC-MS/MS

Abscisic acid (ABA) extraction was conducted following Gómez-Cadenas et al. (2002) with minor modifications (Methods S2). ELISA and UHPLC-MS/MS were used to quantify the endogenous ABA content. ELISA is a rapid and usually sensitive method involved a color intensity measurement and has been reported in the ABA determination (Weiler, 1982; Hirsch et al., 1989). For ELISA determination, the ABA contents were measured using Plant Hormone ABA ELISA Kit (QiYi Biotech, Shanghai, China) according to the manufacturer's protocol.

LC-MS/MS has been applied as an effective method for ABA analysis in plants (Hou et al., 2008; Fan et al., 2011). The combination of UHPLC with tandem mass spectrometry (MS/MS) provides significant advantages in terms of selectivity, sensitivity, and time saving (Romero-González et al., 2008). For UHPLC-MS/MS analysis, the stock solution of ABA standard (purity >98.5%, Sigma-Aldrich, Steinheim, Canada) was dissolved in methanol with a concentration of 1 mg mL^−1^ and stored at −20°C. Working standard solutions were prepared by diluting stock solutions with 80% aqueous methanol prior to use. UHPLC-MS/MS quantification were performed according to Fan et al. (2011) with minor modifications (see Methods S2 for more details).

## Results

### Morphology of resting cysts and vegetative cells of *Scrippsiella trochoidea*

**Figure 1:**
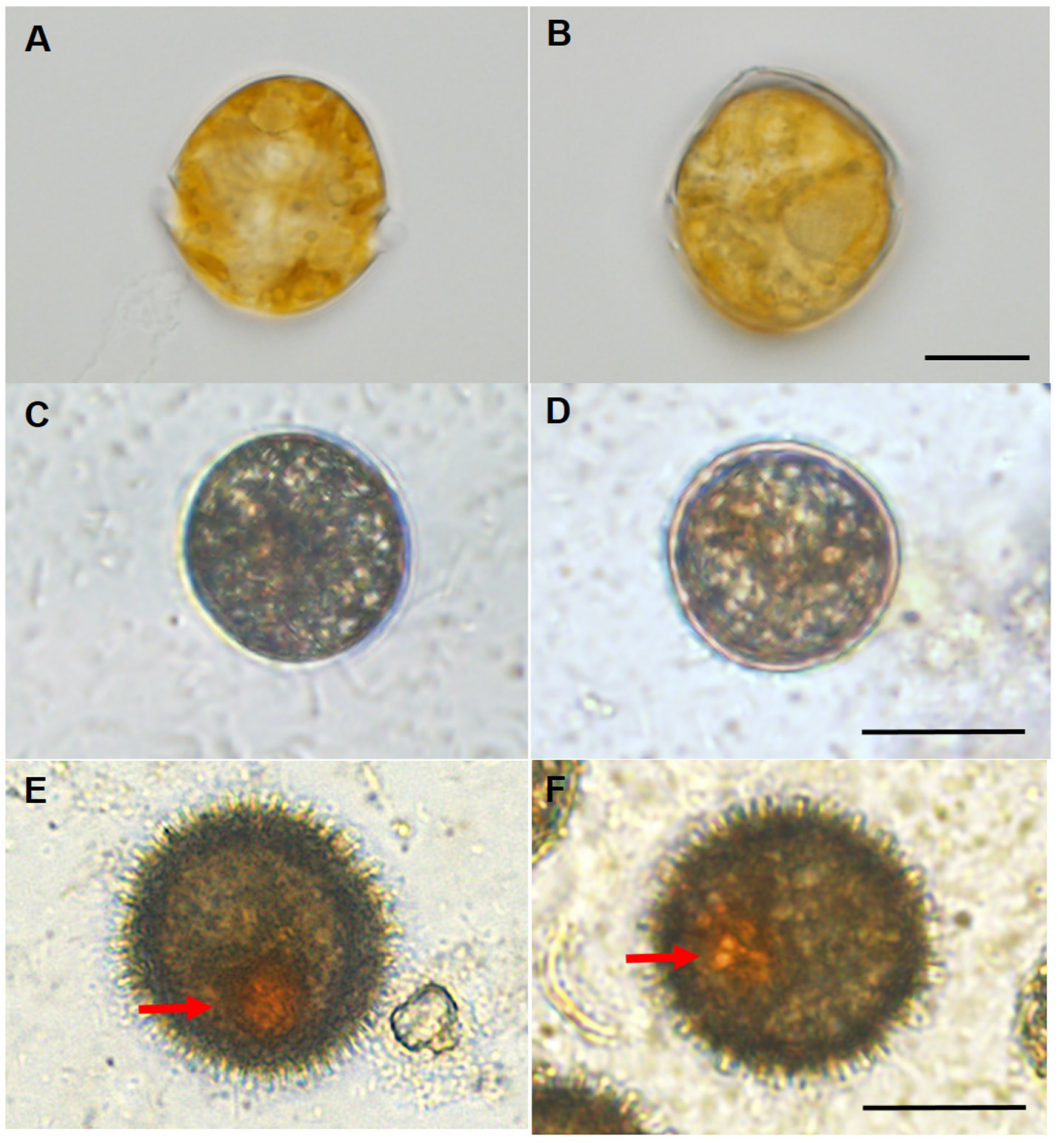
Light microscopic observations for the different morphology of the vegetative cells and resting cysts of *Scrippsiella trochoidea*. **(A,B)** A typical swimming (with two flagella) vegetative cell with conical epitheca, round hypotheca, and apical process. The vegetative cells, immature cysts, and mature cysts could be clearly distinguished from morphological features under light microscope, Scale bar = 10 μm; **(C,D)** immature cysts (sampled from ~40-day-old culture) with a circular shape, without flagellum (thus not swimming), red body and spine. Scale bar = 20 μm; **(E,F)** egg or oval shaped mature resting cysts (sampled from 60-day-old culture) with a red accumulation body (arrows) and numerous surface spines. Scale bar = 20 μm.

The vegetative cells and resting cysts of *Scrippsiella trochoidea* differed considerably as seen from Figure 1, which allowed a swift judgment and separation via light microscopy between the two types of cells. The immature cysts generally appeared circular without red body and spine and were sampled from ~40-day-old cultures (Figures 1C,D); while the mature resting cysts were usually egg or oval shaped with a red accumulation body inside and numerous surface spines, and were sampled from 60 day-old cultures (Figures 1E,F) Vegetative cells swim with their two flagella while resting cysts settled at the bottom (no flagellum) (Figure 1).

### Transcriptome, functional annotation, and qPCR reference genes validation

A total of 358,276,628 raw reads, corresponding to 28.39 Gb of raw data, were generated (Data S1), which were deposited in the NCBI Short Read Archive (SRA) database with the accession number SRP058465. After removing adapters, ambiguous nucleotides, poor quality sequences, and 390 sequences of potentially bacterial contaminnts, ~315.47 million clean reads remained with a mean GC content of 60.97% and an average Q20 percentage of 97.44% (Data S1). Assembly of clean reads finally yielded 166,575 non-redundant unigenes with a N50 length of 1,485 bp and an average size of 969 bp, which lengths ranging from 200 to 12,566 bp (Data S1). The size of current transcriptome is consistent with previously reported transcriptomes of dinoflagellates ranging from ~49 to 191 K, also obtained via RNA-seq method and *de novo* assembly (Zhang et al., 2014; Xiang et al., 2015; Cooper et al., 2016; Guo et al., 2016). All subsequent analyses were based on these non-redundant unigenes.

The non-redundant unigenes were blasted using the non-redundant protein sequence database (NCBI Nr), Kyoto Encyclopedia of Genes and Genomes database (KEGG), Go ontology (GO), Cluster of Orthologous Groups database (COG), and Swissprot databases and 100,788 unigenes, accounting for 60.36% of the total, showed successful blast hits against known sequences in at least one of the abovementioned databases (Data S2). Despite selection of polyadenylated transcripts used to capture primarily eukaryotic Poly-(A)-mRNA (Methods S1), some prokaryotic ribosomal RNAs (rRNA) were detected in the transcriptome assembly (~0.23%). Among the 97,407 hits well annotated with Nr, 5,531 unigenes (5.68%) showed their highest similarities to genes characterized from 55 species (isolates) of dinoflagellates, while the others showed highest similarities to entries from other organisms, particularly other algae and higher plants.

In addition, using the primer sets designed on the basis of the sequence data, expression stabilities of 15 candidate genes were assessed over cultures of *S. trochoidea* at different stages of growth and life cycles (vegetative cells, resting cysts harvested from different time points after formation, and cysts stored at different conditions; see Data S3 for more details). These genes include most of the housekeeping genes (HKGs) that have been used as reference genes in qPCR analyses of algal species (Deng et al., 2016). The combination of *MDH* (malate dehydrogenase), *LBP* (luciferin-binding protein), and *UBC* (ubiquitin conjugating enzyme) was found to be the best (i.e., most stable) for the purpose of normalizing expression levels (Data S3). The result was then used in our qPCR analyses (see below).

### Global changes of gene expression in resting cysts

In total, 3,874 unigenes were identified to be DEGs between resting cysts and vegetative cells, with 2,465 being up-regulated and 1,409 down-regulated (Table S1). Of the 3,874 DEGs, 1,914 unigenes were categorized into 118 pathways according to KEGG annotations (Table S2). Pathway analysis of those annotated DEGs showed that they were mainly involved in transcription, transport and catabolism, carbohydrate metabolism, lipid metabolism, amino acid metabolism, environmental adaptation, and others (Table S2). Among them, 2 pathways, “base excision repair” (20; 1.04%) and “valine, leucine and isoleucine degradation” (16; 0.84%) were significantly enriched (*p* ≤ 0.05).

Notably, 134 unigenes, with 41 having well defined functions, were detected in the transcriptomes of resting cysts only (Table S11). The same expression changes between vegetative cells and resting cysts as seen from the transcriptomes were further confirmed with qPCR analyses for 3 unigenes (Unigene31065, Unigene10081, and Unigene53202) (Figures 6Aa,Ba,Ca). The 41 well-annotated DEGs covered a wide range of functions, including gene regulation (e.g., transcriptional regulators), RNA regulation, protein degradation, material transport, and membrane organization (Table S11).

### Genes potentially involved in sexual reproduction and encystment

Based on annotations, we identified 284 unigenes being potentially involved in sexual reproduction and cyst formation (annotation categories “spore formation and germination” and “sexual reproduction”), among which 12 displayed differential expressions between vegetative cells and resting cysts (Table S3). The 10 sequences out of the 12 DEGs showing as orthologues to genes encoding members of MEI2-like family, genes regulating meiosis in sexual reproduction of eukaryotes, with 6 of them down-regulated in resting cysts; the other 2 DEGs were orthologues of genes encoding SPO11-2 (recombination protein SPO11-2) and DMC1 (recombination protein DMC1) (Table S3).

### Genes controlling metabolism of ABA, their expression patterns, and direct measurements of ABA in vegetative cells and cysts via ELISA and UHPLC-MS/MS

The phytohormone ABA has been shown to play vital roles in both the establishment and maintenance of seed dormancy in higher plants (Koornneef et al., 2002; Kucera et al., 2005; Nambara and Marion-Poll, 2005; Footitt et al., 2011). The enzymes involved in ABA synthesis and catabolism, NCED (catalyzing the rate-limiting step of ABA synthesis), ZEP (accounting for the first step of ABA synthesis), AAO (responsible for the final step of ABA synthesis), and ABAH (catalyzing the rate-determining step of ABA catabolism), are established to be key factors regulating cellular ABA content. In this study, we identified 9 unigenes as *NCED*s, 15 *ZEP*s, 2 *AAO*s, and 12 *ABAH*s in *S. trochoidea* vegetative cells and cysts (Table S6). Based on the sequences from the transcriptomes, we then obtained, via RACE-PCR, the full-length cDNA sequence for each of an *ZEP, NCED, AAO*, and *ABAH* gene from *S. trochoidea* (designated as *StZEP, StNCED, StAAO*, and *StABAH*, GenBank accession Nos. KT033705, KR148942, KR148943, and KR148944, respectively) (Table S4). Their respective expressions in cells at different stages of growth and resting cysts with different durations of dormancy and different storage temperatures were then tracked via qPCR.

**Figure 2:**
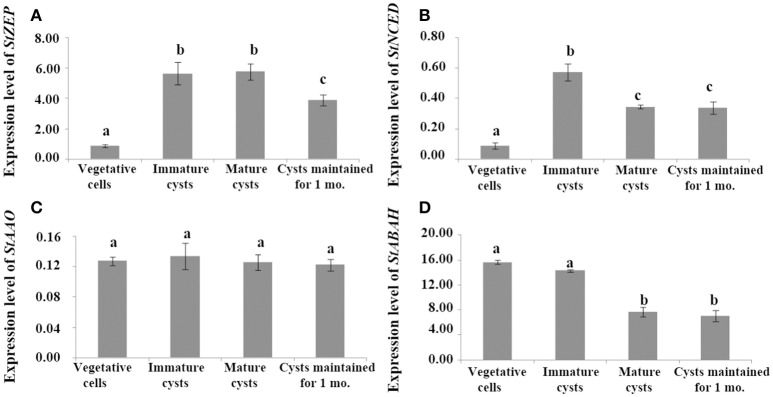
The transcript levels of genes associated with ABA biosynthesis and catabolism, **(A)** *StZEP* **(B)** *StNCED* **(C)** *StAAO* **(D)** *StABAH*, relative to the reference gene *MDH* in cells at different stages of life cycle (vegetative cells, immature cysts, mature cysts, and cysts maintained for 1 month). Significant differences in abundance are indicated with different letters above bars at *p* < 0.05; same letter denotes no significant difference. Values are presented as mean ± standard deviation, *Error Bars* = SD, *n* = 3.

Comparing expressions between vegetative cells and cysts, *StZEP* expressions in immature and mature cysts were significantly higher than that in vegetative cells (ANOVA, *p* < 0.01), and although the expression declined in cysts maintained for 1 month, it still remained significantly higher than that in vegetative cells (ANOVA, *p* < 0.01, Figure 2A and Data S4); *StNCED* expressions were also higher in cysts (including immature cysts, mature cysts, and cysts maintained for 1 month) and reached a peak in immature cysts, which was >1.5-fold higher than that in all other stages (ANOVA, *p* < 0.01, Figure 2B and Data S4); *StAAO* expressions were not significantly different between cysts and vegetative cells (ANOVA, *p* > 0.05, Figure 2C and Data S4); *StABAH* expressions were significantly higher in vegetative cells and immature cysts than those in mature cysts and cysts maintained for 1 mo. (ANOVA, *p* < 0.05, Figure 2D and Data S4).

**Figure 3:**
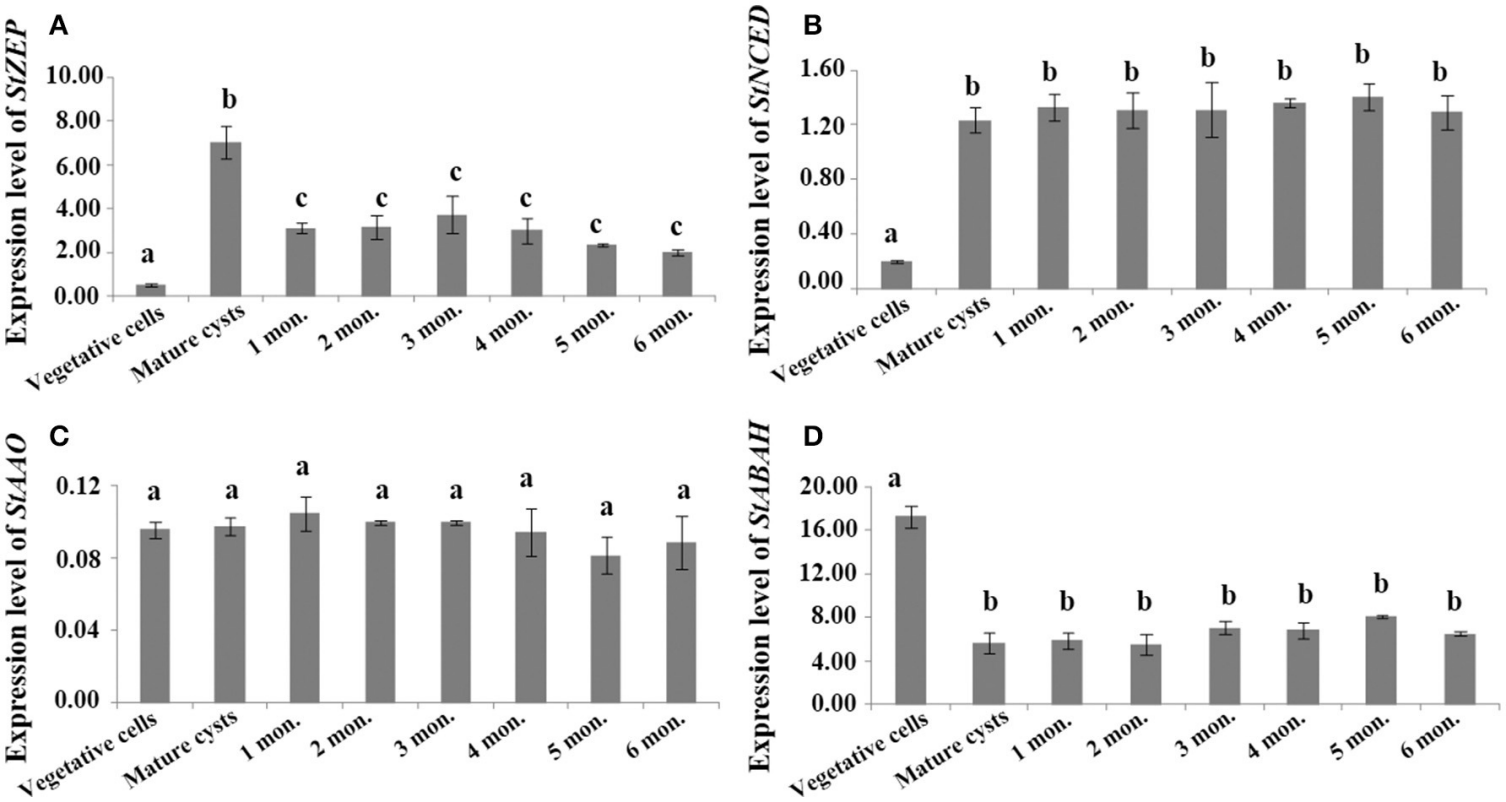
The transcript levels of genes associated with ABA biosynthesis and catabolism, **(A)** *StZEP* **(B)** *StNCED* **(C)** *StAAO* **(D)** *StABAH*, relative to *MDH* (reference gene) in resting cysts maintained at 20 ± 1°C for different periods of incubation time (0–6 month). The Y axis shows the expression levels of targeted genes relative to the reference gene; Significant differences in abundance are indicated with different letters above bars at *p* < 0.05; same letter denotes no significant difference. Values are presented as mean ± standard deviation, *Error Bars* = SD, *n* = 3.

**Figure 4:**
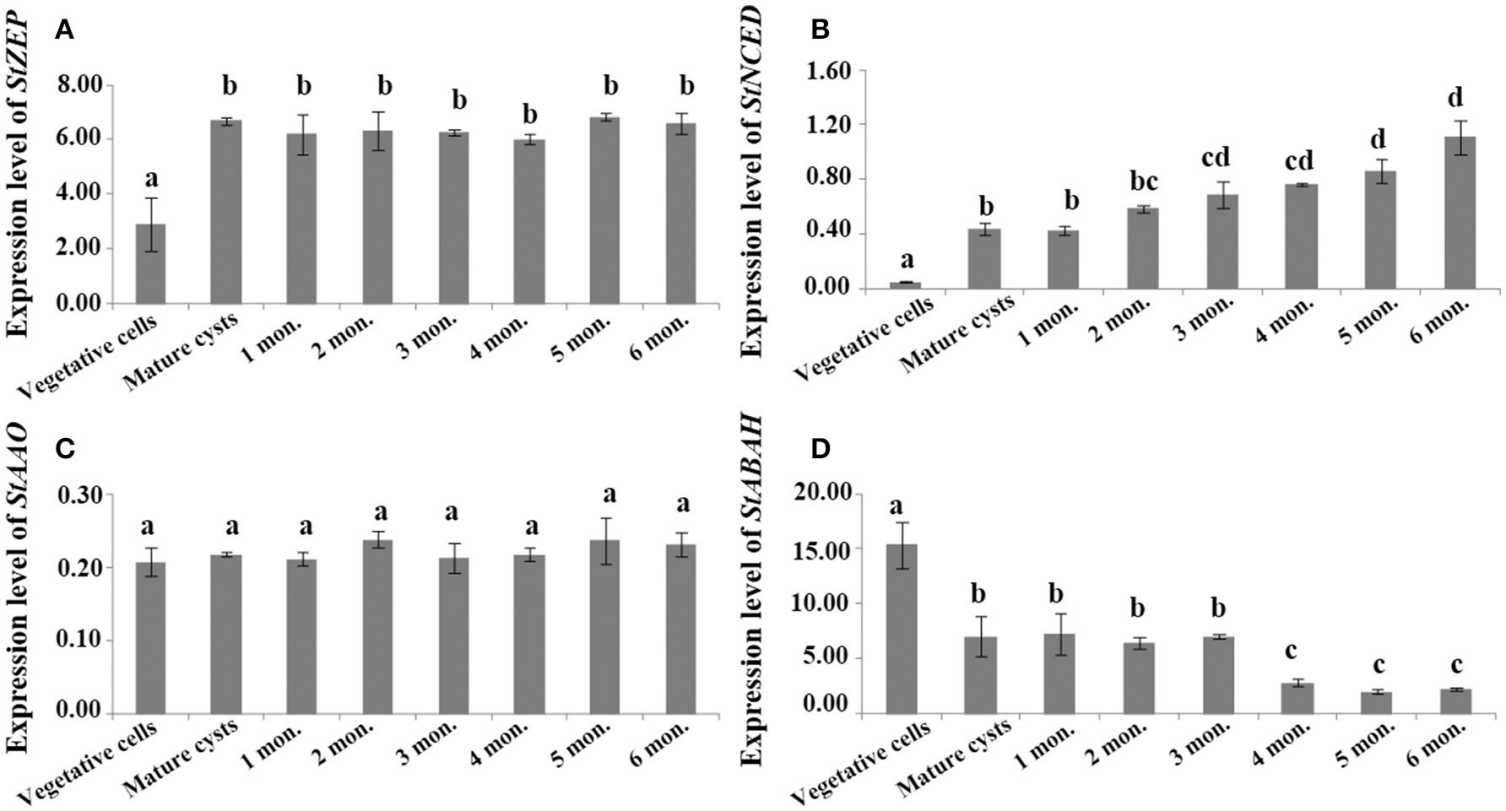
The transcript levels of genes associated with ABA biosynthesis and catabolism, **(A)** *StZEP* **(B)** *StNCED* **(C)** *StAAO* **(D)** *StABAH*, relative to *MDH* (reference gene) in resting cysts stored at 4 ± 1°C in darkness for different periods of time (0–6 month). The Y axis shows the expression levels of targeted genes relative to the reference gene; Significant differences are presented with different letters above bars at *p* < 0.05; same letter denotes no significant difference. Values are presented as mean ± standard deviation, *Error Bars* = SD, *n* = 3.

For cysts maintained at routine culturing conditions, *StZEP* expressions were observed significantly higher in newly matured cysts (5–13-fold higher) than that in vegetative cells, and then decreased with the time of storage from 1 to 6 mo., but still statistically higher than that in vegetative cells (ANOVA, *p* < 0.05, Figure 3A and Data S5); *StNCED* expressions showed a similar significant increase in newly matured cysts and maintained the level for 6 month of storage; *StABAH* expressions displayed a significant decrease from vegetative cells to newly matured cysts and then maintained the low level for 0–6 mo. (ANOVA, *p* < 0.01, Figures 3B,D and Data S5); *StAAO* expressions showed no significant difference between vegetative cells and cysts and among cysts with different time of storage (ANOVA, *p* > 0.05, Figure 3C and Data S5). For cysts maintained in dormancy at 4 ± 1°C in darkness (to simply imitate the natural conditions of marine sediment) for 0–6 months, *StZEP* and *StNCED* expressions were relatively high, with no significant change in *StZEP* and a gradual increase in *StNCED* during 6 mo. dormancy (ANOVA, *p* > 0.05, Figures 4A,B and Data S6); *StAAO* expressions were statistically the same as that in vegetative cell control throughout the 6 mo. dormancy (ANOVA, *p* > 0.05, Figure 4C and Data S6); *StABAH* expressions was strongly suppressed during the whole period of dormancy and declined to a lower level after 3 mo. (ANOVA, *p* < 0.05, Figure 4D and Data S6).

**Figure 5:**
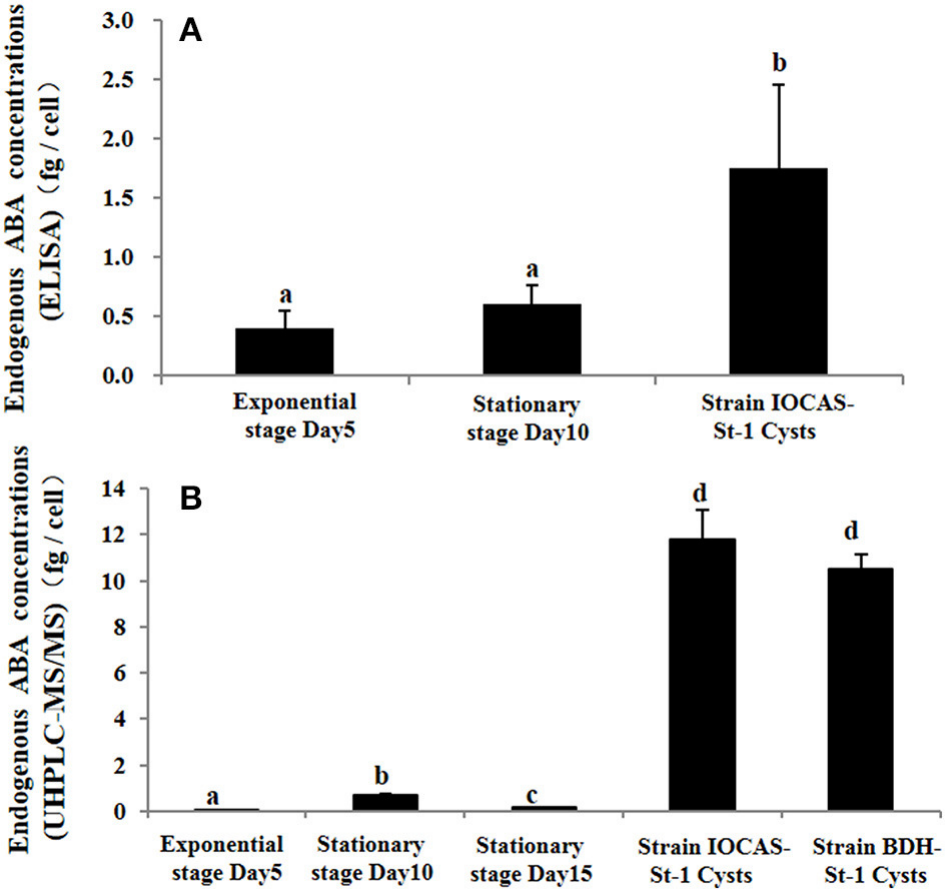
Endogenous ABA concentrations in vegetative cells (at exponential and stationary growth stages) and resting cysts of *S. trochoidea* via **(A)** ELISA and **(B)** UHPLC-MS/MS quantification. Growth stages of strain IOCAS-St-1 were determined according to the growth curve (Supporting information Methods S2). The Y axis shows the expression levels of targeted genes relative to the reference gene; Significant differences are denoted with different letters above bars at *p* < 0.05; same letter denotes no significant difference. Values are presented as mean ± standard deviation, *Error Bars* = SD, *n* = 3.

Then endogenous ABA contents in resting cysts and vegetative cells were both measured using ELISA and UHPLC-MS/MS. For ELISA determinations, markedly higher ABA concentration (~3.5-fold) was detected in mature resting cysts than that in vegetative cells (ANOVA, *p* < 0.01, Figure 5A), ABA concentrations in vegetative cells at exponential and stationary stages were not significantly different from each other (ANOVA, *p* > 0.05, Figure 5A). For UHPLC-MS/MS quantification, the ABA concentration in mature cysts was detected to be ~39-fold of that in vegetative cells (ANOVA, *p* < 0.001, Figure 5B). Higher ABA (UHPLC-MS/MS: ~39-fold; ELISA: ~3.5-fold) was measured in vegetative cells at stationary stage than that at exponential stage (Figure 5).

### Genes related to phytohormones signal transduction

**Figure 6:**
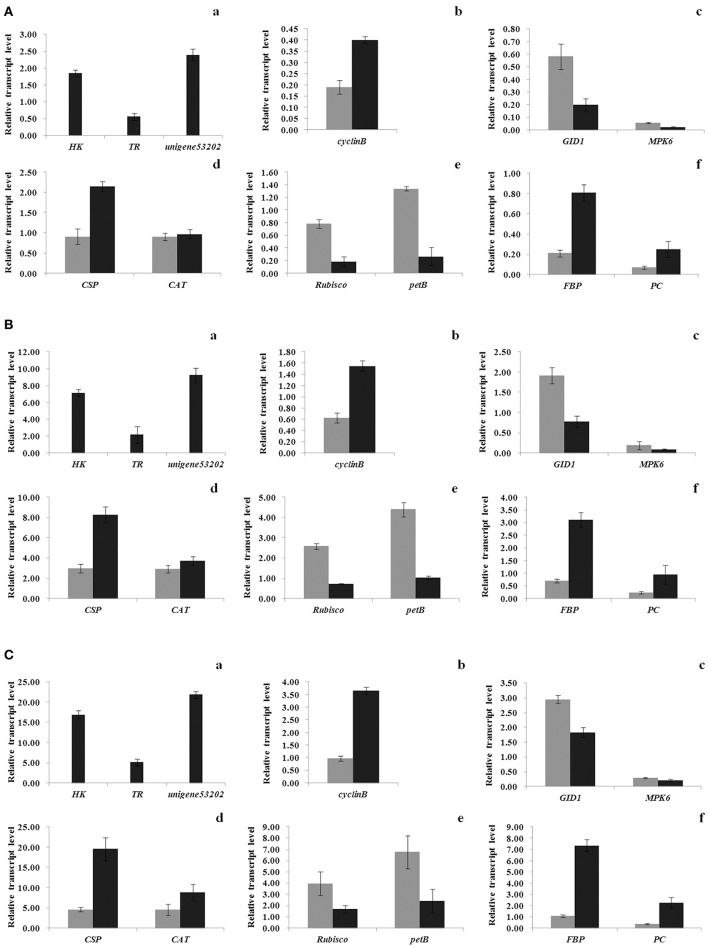
Verification of expression levels for selected unigenes from the RNA-seq via qPCR. Total RNA was extracted from vegetative cells and resting cysts of *Scrippsiella trochoidea*, respectively. Relative expression levels of unigenes are presented as the expression normalized to the reference gene **(A)** *MDH*, **(B)** *LBP*, and **(C)** *UBC*, resectively. For each reference gene, the expression levels are presented for unigenes (a) specifically expressed in resting cyst, (b) relevant to cell cycle, (c) relevant to phytohormones signal transduction, (d) relevant to environmental adaptation, (e) relevant to photomorphogenesis, and (f) relevant to energy metabolism. For each gene, the gray and black bars indicate the gene expression for vegetative cells and resting cysts, respectively. Values are mean ± standard deviation, *Error Bars* = SD, *n* = 3.

In addition to ABA, other well-known phytohormones were also detected in the transcriptome of *S. trochoidea*: 648 unigenes were found to be homologous to the genes associated with hormones in higher plants, which are essential components in signaling systems of phytohormones, including auxin, ABA, cytokinin (CK), ethylene (ET), gibberellin (GA), brassinosteroid (BR), jasmonic acid (JA), and salicylic acid (SA) (Table S5, Figure S1). In resting cysts, 6 of the abovementioned unigenes significantly increased their expressions, 15 significantly decreased, and these 21 DEGs were predicated being relevant to signal transductions of ABA, CK, ET, GA, BR, and SA (Table S5; Figure S1). The same direction in expression change for 2 of them, a putative gibberellin receptor GID1 (Unigene78216) and a putative mitogen-activated protein kinase 6 (MPK6) (Unigene555), were further verified by qPCR analyses (Figures 6Ac,Bc,Cc).

### DEGs relevant to cell cycle

Since resting cyst is a dormant stage in the life history of dinoflagellate, and encystment generally leads to a suspension of cell division, we assumed that the genes regulating cell cycle may be also linked to resting cyst formation. Cyclin/CDK (cyclin dependent kinase) complexes, are central in the regulation of cell cycle in eukaryotes as they govern the entry into S and M phases (King et al., 1994; Lee et al., 2003). We obtained 87 and 57 transcripts encoding the CDK and cyclin family genes in *S. trochoidea*, respectively. Among them, one putative B-type CDK (CDK-B; Unigene54695) and one predicted F-type CDKs (CDK-F; CL5753.Contig2) greatly decreased their expressions in resting cysts, while one B-type cyclin (Unigene51485) and one U-type cyclin (Unigene99561) strongly elevated their expressions in resting cysts (Table S7).

### DEGs linked to photosynthesis and direct measurements of photosynthesis efficiency

We identified 293 transcripts encoding subunits of photosynthetic multi-subunit membrane-protein complexes including photosystem I (PSI), photosystem II (PSII), cytochrome *b*_6_/*f* complex, and F-ATPase, all essential in catalyzing oxygenic photosynthesis (McCarty et al., 2000; Kallas, 2004; Nelson and Yocum, 2006; Yang et al., 2015). Among them, 11 sequences, including those encoding for basic components in PSI [PSI chlorophyll *a* apoprotein A1 (PsaA), PSI chlorophyll *a* apoprotein A2 (PsaB)], PS II [PSII reaction center D1 protein (PsbA), PSII CP47 chlorophyll apoprotein (PsbB), PSII CP43 chlorophyll apoprotein (PsbC), PSII reaction center D2 protein (PsbD), PSII cytochrome b559 subunit alpha (PsbE)], cytochrome *b*_6_/*f* complex (cytochrome *b*_6_ and subunit IV), and F-type ATPase (F1-ATPase subunit α, β), showed strikingly reduced expressions in resting cysts (Figure S2 and Table S8). We noted 2 DEGs encoding for ribulose-1, 5-bisphosphate carboxylase/oxygenase (Rubisco) also significantly down-regulated in resting cysts (Table S8), and the repressed expression for one of them (Unigene20500) was further confirmed in the qPCR experiment (Figures 6Ae,Be,Ce). Also, we measured the apparent PSII photochemical quantum efficiency (*F*v/*F*m) in vegetative cells and resting cysts and observed that only minimal *Fv*/*Fm*-value was detected in resting cysts in comparison to that in vegetative cells (0.033 ± 0.0045 of resting cysts vs. 0.543 ± 0.0096 of vegetative cells, ANOVA, *p* < 0.01, Figure S3).

### DEGs associated with environmental adaptation

Encystment is widely perceived as an adaptive strategy for dinoflagellates to survive unfavorable conditions and resting cysts are also considered to endure challenging conditions of temperature, darkness, grazers or parasite attacks, and other abiotic or biotic stresses before germination. In our transcriptomes, 45 DEGs were recognized to be relevant to environmental adaptation processes, including 21 encoding enzymes belonging to the reactive oxygen species (ROSs)-scavenging system (including ascorbate peroxidase, APX; monodehydro-ascorbate reductase, MDHAR; glutaredoxin, Grx; thioredoxin, Trx; and catalase, CAT); 2 encoding cold shock proteins (CSPs); 3 encoding heat shock protein 70s (Hsp70s); and 19 encoding for chitin deacetylase, chitinases, or β-1, 3-glucanases (GLUs), which are enzymes essential for pathogens resistance (Table S9). As a general pattern, while the majority of them (28 out of 45 DEGs) were down-regulated in resting cysts, 13 of the 21 DEGs encoding ROS-scavenging enzymes and the one encoding chitin deacetylase were up-regulated (82% of the 17 up-regulated genes). The same trend in expression change observed from the assembly for 2 of the ROS-scavenging enzyme-relevant DEGs (CSP and CAT) were further verified with qPCR experiments (Figures 6Ad,Bd,Cd).

### DEGs relevant to energy metabolism and direct measurements of respiration rates in vegetative cells and resting cysts

A total of 45 DEGs in *S. trochoidea* were identified to be relevant to energy metabolism, which include categories of glycolysis, citrate cycle (TCA cycle), glyoxylate pathway, and fatty acid metabolism (Table S10). The 37 DEGs encoding enzymes for glycolysis and TCA cycle include fructose-1, 6-bisphosphatase (FBP), phosphofructokinase (PFK), aldolase, phosphoglycerate mutase, glyceraldehyde 3-phosphate dehydrogenase, phosphoglycerate kinase, enolase, pyruvate kinase (PK), aldehyde dehydrogenase, alcohol dehydrogenase, dihydrolipoamide acetyltransferase, pyruvate carboxylase (PC), dihydrolipoamide dehydrogenase, aconitase, and succinate dehydrogenase. The The same trend in expression of 2 unigenes, Unigene107654 and Unigene20189, were verified via qPCR to be dramaticly up-regulated in resting cysts by 3.8–6.9- and 3.6–6.5-fold, respectively (Figures 6Af,Bf,Cf). Also, 7 DEGs encoding components for fatty acid metabolism, long-chain acyl-CoA synthetases (LACS), enoyl-CoA hydratase, acetyl-CoA acyltransferase, and acetyl-CoA C-acetyltransferase, as well as 1 DEG encoding malate synthase (MS) for glyoxylate pathway were observed in resting cysts (Table S10). By measuring oxygen consumptions, the calculated respiration rates in resting cysts were significantly lower than that in vegetative cells but with a detectable level (0.002 ± 0.0006 vs. 0.009 ± 0.001, ANOVA, *p* < 0.01. Figure S4).

## Discussion

### General transcriptome features and global changes of gene expression in resting cysts

Our *de novo* assembly reconstructed 166,575 non-redundant unigenes, a size consistent with but slightly higher than that reported in the literature for dinoflagellates (~49–191 K transcripts; Zhang et al., 2014; Xiang et al., 2015; Cooper et al., 2016; Guo et al., 2016). Up to 69,558 unigenes (41.75%) did not have similarity to any sequence in these databases, a number similar to that of the most recently published transcriptome of the species (Cooper et al., 2016) and that of other dinoflagellates obtained via RNA-Seq (Zhang et al., 2014; Xiang et al., 2015; Guo et al., 2016), and were at least partly caused by highly limited studies on functional genes and enormous genome sizes of dinoflagellates. The 3,874 DEGs (2.32% of total unigenes) that differentially expressed between resting cysts and vegetativ cells of *S. trochoidea* are more than that recorded in the dinoflagellate *Lingulodinium polyedrum* (0.18% DEGs between vegetative cells and cold-induced temporary cysts; Roy et al., 2014). Our results indicate that numerous genes are still actively expressed in resting cysts although resting cysts of dinoflagellates are generally perceived as being in physiological dormancy.

To exclude prokaryotic RNA, we first tried our best to obtain axenic cultures, and then used oligo-(dT) conjugated magnetic beads (Illumina, USA) to purify poly-(A)-containing mRNA from total RNA. However, a small amount of prokaryotic rRNA (~0.23%) were still detected in the transcriptome assembly. Other omics investigations on dinoflagellates were also concerned with the same problem (Lin et al., 2015; Xiang et al., 2015; Cooper et al., 2016; Guo et al., 2016). The most likely explanation is that many dinoflagellates are hosts of endosymbiotic bacteria (Lin, 2011), which makes obtaining axenic cultures extremely difficult. In addition, some bacteria are resistant to multiple antimicrobials (Dang et al., 2008, 2009), thus the antibiotics used in this study might not eliminate or inhibit all the bacteria in the dinoflagellate cultures. The third explanation, likely a very important one, is related to algal surface-associated bacteria. Many bacteria can be surface-attached and this lifestyle presents certain ecophysiological advantages to the colonizing bacteria, including protection from antibiotics and other harmful physicochemical and biological effects (Dang and Lovell, 2016).

### Genes potentially pertinent to sexual reproduction and encystment

Encystment of dinoflagellates is generally accompanied by a series of cellular processes such as gamete formation, sexual mating, cell fusion, and cell morphogenesis (Bravo and Figueroa, 2014; Tang et al., 2016). We compiled 284 sequences pertinent to sexual reproduction and cyst formationin *S. trochoidea*, with 12 of them displayed differential expressions between vegetative cells and resting cysts (e.g., *MEI2, SPO11*). In the yeast *Schizosaccharomyces pombe*, the *MEI2* gene is the master regulator for switching from mitosis to meiosis and encodes an RNA binding protein required for premeiotic DNA synthesis and entry into meiosis I (Jeffares et al., 2004; Kaur et al., 2006). Bioinformatic study indicated MEI2-like genes are widespread in the plant kingdom (Jeffares et al., 2004). Our results confirmed their presence in dinoflagellates and suggested the 4 up-regulated and 6 down-regulated MEI2-like unigenes are probably involved in the sexual reproduction and encystment of *S. trochoidea*. However, since meiosis of vegetative cells prior to formation of gametes and fusion of gametes has never been observed during encystment of dinoflagellates, the currently available evidence does not allow us to speculate further upon the exact roles played by these genes in the encystment and dormancy of *S. trochoidea*. Many plant species have 3 homologs of SPO11. In *Arabidopsi*s, SPO11-2 functions with SPO11-1 in meiotic recombination, while SPO11-3 functions in DNA replication (Stacey et al., 2006). DMC1 is a meiosis-specific gene of the *Escherichia coli* recombination protein RecA, which is required for meiotic recombination, synaptonemal complex formation, and progression out from meiotic prophase (Bishop et al., 1992). In this study, expressions of SPO11-2 and DMC1 unigenes in resting cysts were both significantly higher than that in vegetative cells, suggesting that they were likely related to meiotic recombination and sexual and dormancy processes in resting cysts of *S. trochoidea*.

Molecular analyses of events leading up to meiosis and spore formation in plants have primarily been advanced upon studies on *Arabidopsis thaliana* and *Zea mays*. Among the compiled 284 unigenes associated with sexual reproduction and cyst formation in the assembled *S. trochoidea* transcriptome, some of their homologs were also detected in the genomes and transcriptomes of dinoflagellate *Symbiodinium* species (Bayer et al., 2012; Shoguchi et al., 2013; Chi et al., 2014; Lin et al., 2015; Levin et al., 2016). Previously, 6 meiosis-specific and 25 meiosis-related genes were demonstrated in published genomes of *Symbiodinium* species (Bayer et al., 2012; Shoguchi et al., 2013); and cryptic sex is suspected to occur in *Symbiodinium*'s seldom-seen free-living state (Chi et al., 2014). Collectively, these results indicate that, while many genes encoding basic components of the meiotic machinery are conserved among dinoflagellates and higher plants, other unigenes that could not be annotated (i.e., have no counterparts in higher plants) may be unique to dinoflagellates.

### ABA as a possible regulator of encystment and dormancy in dinoflagellate

Endogenous ABA concentrations are the result of a dynamic balance between continuous synthesis and catabolism (Cutler and Krochko, 1999), and, therefore, the active hormone level that controls physiological processes results from changes in either synthesis or catabolism (Nambara and Marion-Poll, 2005). Tremendous progress has been made in the molecular mechanisms in higher plants, and some enzymes involved in synthetic and catabolic pathways, such as NCED, ZEP, AAO, and ABAH, were shown to be key determinants in regulation of cellular ABA content. In this study, we detected the presence and obtained the full-length cDNA sequences of these genes. Our qPCR results clearly indicated that the elevated ABA biosynthesis was concurrent with the transformation from vegetative cells into resting cysts, which is in accordance with the increased amount of ABA in seeds of higher plants during dormancy. For resting cysts with different durations of dormancy and different storage temperatures, the qPCR results demonstrated an elevated biosynthesis and repressed catabolism of ABA during the courses of encystment and cyst dormancy, which was significantly enhanced by lower temperature and darkness.

In order to test whether the expression levels of these 4 ABA-relevant genes were in fact linked to the cellular ABA levels, we measured the ABA contents in vegetative cells and cysts using ELISA; and then for more accurate measurement, we conducted UHPLC-MS/MS quantification. We did repeatedly observe significantly higher contents of ABA in mature resting cysts than that in vegetative cells at exponential and stationary stages from both UHPLC-MS/MS (~39-fold) and ELISA (~3.5-fold) measurements. These direct measurements of ABA were highly consistent with the above-described qPCR results. Considering the role of ABA in higher plants, the most parsimonious explanation for these measurements is that ABA is required for resting cysts to maintain dormancy. Collectively, our results strongly suggest that *StZEP, StNCED*, and *StABAH* were involved in regulating endogenous ABA level in *S. trochoidea* and pointed to a possibly vital regulatory role of endogenous ABA in resting cyst formation and dormancy maintenance.

### Phytohormones signal transduction

Phytohormone systems generally include biosynthesis pathways and signal transduction pathways that mediate the effects of phytohormones. In our data, 648 unigenes were found to be homologous to the known hormones that are essential components in signaling systems of phytohormones in higher plants, including auxin, ABA, CK, ET, GA, BR, JA, and SA. However, many components for those pathways established from higher plants were missing in our transcriptomes. A recent review on genome-based metabolic reconstructions in different microalgae (including stramenopiles, archaeplastida, and cyanophytes) proposed that microalgae and higher plants shared most of the key components in phytohormone signaling circuitries, but other presently unknown mechanisms for transmitting phytohormone signals may exist in microalgae (Lu and Xu, 2015). Our results support this point, while it is also possible that our transcriptomes might not be fully inclusive.

In higher plants, a number of phytohormones play vital roles in the modulation of seed dormancy and germination (Koornneef et al., 2002; Footitt et al., 2011; Kiseleva et al., 2012). While ABA has been well documented in inducing seed dormancy, GA regulates dormancy release and germination of seeds (Brady and McCourt, 2003; Kucera et al., 2005). A dynamic balance of the ABA and GA is thought to be crucial in controlling dormant status, while other hormones may also influence the process through the ABA/GA ratio (Footitt et al., 2011). BR and ET counteract the inhibitory effects of ABA on seed germination, but in most species, they act after dormancy has been released by GA (Brady and McCourt, 2003; Footitt et al., 2011); CK appears to promote dormancy release and subsequent germination by enhancing ET biosynthesis (Koornneef et al., 2002; Kucera et al., 2005); SA exerts an antagonistic effect on GA-induced seed germination (Xie et al., 2007). In *S. trochoidea*, 21 sequences of the abovementioned 648 genes greatly altered their expressions in resting cysts, which were associated with signal transductions of ABA, CK, ET, GA, BR, and SA. It appeared that there possibly exists cross-talk among phytohormones in regulating resting cyst formation and dormancy in *S. trochoidea*, which guarantees future in-depth investigations for clarification. Nevertheless, our results provided fresh insights into the regulatory networks of phytohormones in dinoflagellates and a basis for future identification of signaling components and specific functional analyses in the vast and phylogenetically diverse microalgae.

### Cell cycle

Dinoflagellates have been established to follow the typical eukaryotic cell cycle regulation scheme, and the oscillation in the sequential activation and deactivation of CDKs, which is triggered by binding to their specific regulatory subunits (i.e., cyclins), achieve appropriate responses of cell division to intrinsic and extrinsic signals (Wang D. Z. et al., 2013). Our data set identified 87 and 57 unigenes encoding the CDK and cyclin family members, respectively, of which 2 putative CDKs exhibited decreased expressions in resting cysts. One of them (Unigene54695) is homologous to members of plant specific CDK-B family, whose activity is prominently linked to mitosis and transcripts accumulation during G2/M transition (Lee et al., 2003; Bisova et al., 2005). Another sequence (CL5753.Contig2) encoding putative CDK-F, which is CDK-activating kinases (CAK) serving to activate A-type CDKs and function without any binding partner (Bisova et al., 2005). Our results suggest they seemed to be involved in (or, parallel to) the encystment or dormancy maintenance of *S. trochoidea*. In addition, 2 putative cyclins (a putative cyclin B, Unigene51485, and a homolog of the rarely reported cyclin U, Unigene99561) elevated their expressions in resting cysts, as seen both in RNA-seq and qPCR detection Figures 6Ab,Bb,Cb. The mitosis-associated cyclin B accounts for the transition from G2 to M phase, and accumulates in G phase but falls in the rest of cell cycle in many eukaryotes (King et al., 1994). Cyclin U was preliminarily documented to play roles in mediating cell division to control leaf erectness in *Oryza sativa* (Sun et al., 2015). The presence of U-type cyclin in *S. trochoidea* and its up-regulated expression in resting cysts imply that Unigene99561 may be associated with the dormancy of resting cysts.

One may reasonably postulate that genes regulating cell cycle may be also linked to resting cyst formation, since encystment generally leads to a suspension of cell division. However, we failed to find any DEG in our KEGG pathways by searching with the term “cell cycle,” which was in agreement with the absence of transcriptional control of cell-cycle pathway in the dinoflagellate *Karenia brevis* (Van Dolah et al., 2007; Brunelle and Van Dolah, 2011). In *Karenia brevis*, most of the cell cycle genes appeared to be post-transcriptionally regulated, which was supported with the discovery of spliced leader genes in the species (Zhang et al., 2007; Brunelle and Van Dolah, 2011). In most eukaryotic organisms, circadian output of cell division is exerted primarily over transcriptional modulations; thus, the post-transcriptional control of cell cycle rhythm in dinoflagellates, which represents a novel mechanism, is of particular interest (Van Dolah et al., 2007). Further combined analyses of transcriptome and proteome are anticipated to generate a more comprehensive view in this regard and help to elucidate the underlying links between cell cycle and resting cyst formation.

### Repressed photosynthesis in resting cysts

In our study, all 11 DEGs relevant to photosynthesis were strikingly reduced in abundance in resting cysts, and seven of them putatively encoding basic elements of the photochemical reaction centers were down-regulated in resting cysts by 4–8-fold, clearly indicating a dramatic decrease of light-driven electron-transfer reactions. The cytochrome *b*_6_/*f* complex catalyzes the rate-limiting quinol-oxidation step in the oxygenic photosynthesis (Kallas, 2004). The significantly reduced transcripts of the hypothetic cytochrome *b*_6_ and subunit IV (>5-fold in resting cysts) would affect the cyclic electron flow, and are unfavorable to photosynthetic ATP production. The significantly decreased transcriptions of F1-ATPase subunits α and β in resting cysts should indicate a declined ATPase activity and ATP production. In addition, 2 repressed DEGs encoding for Rubisco, which is the key enzyme responsible for the first step of photosynthetic carbon assimilation and directly determines the photosynthetic rate (Andersson and Backlund, 2008), also provided evidence for inhibition of carbon fixation. Supportingly, we measured significantly lower *Fv/Fm*-value in resting cysts than that in vegetative cells, which indicated a down-regulation of PSII reaction centers or PSII inactivation in resting cysts. Roy et al. (2014) has shown that photosynthesis was down-regulated in the temporary cysts of dinoflagellate *Lingulodinium polyedrum*. Our results here together evidenced clearly a pause of photosynthesis in resting cysts, which is well understandable for cells in dormancy.

### Environmental adaptation

In our data, 44 DEGs was pertinent to environmental adaptation during resting cyst formation and dormancy maintenance of *S. trochoidea*. ROSs (e.g., superoxide, H_2_O_2_, and OH^−^) are produced intracellularly in response to various stressors due to malfunctioning of cellular components (Mittler et al., 2004) and can cause oxidative damage to proteins, lipids, and nucleic acids. Cells possess a battery of antioxidant enzymes to resist oxidative damage (Imlay, 2008; Lillig et al., 2008; Wang J. et al., 2013). The glutathione-ascorbate cycle (GSH-AsA cycle) eliminates H_2_O_2_ and provides endogenous defense against harmful accumulation of ROSs (Mittler et al., 2004). In the cycle, ascorbate peroxidase (APX) uses ascorbic acid (AsA) as an electron donor to oxidize radicals by reducing H_2_O_2_ to water and oxidizing ascorbate acid (vitamin C) to dehydroascorbate, whereas AsA is oxidized into monodehydroascorbate (MDHA). MDHA is then reduced back to AsA by MDHAR (Wang J. et al., 2013). In our DEGs data, 3 APXs (2 up-regulated and 1 down-regulated) and 2 MDARs (1 up-regulated and 1 down-regulated) were detected. These 5 DEGs may thus participate in antioxidant defense of resting cysts via GSH-AsA cycle. Glutaredoxins (Grxs) are small heat-stable disulfide oxidoreductases, which protect cells from H_2_O_2_-induced apoptosis (Lillig et al., 2008). Thioredoxins (Trxs) actively maintain intracellular thiol-redox homeostasis (Holmgren, 1985). Our results contained 3 Grxs (2 up-regulated and 1 down-regulated) and 10 Trxs (6 up-regulated and 4 down-regulated). Catalase (CAT) directly reduces H_2_O_2_ to water and oxygen (Imlay, 2008). Three DEGs in our data were annotated as CAT, 2 of them up-regulated and 1 down-regulated; qPCR analysis further confirmed elevated expression for 1 CAT (Unigene99398) in resting cysts. In general, the up-regulated expressions for most of the genes encoding ROS-scavenging enzymes suggested an elevated necessity of antioxidizing mechanisms for resting cysts at dormancy.

Cold shock proteins (CSPs) are induced upon temperature downshifting and are responsible for acclimation of cells to cold. Apart from cold stress responses, they also present under normal conditions to regulate other biological functions (Sasaki and Imai, 2012). While previous work reported the abundance of CSPs declined in resting cysts of ciliate (Chen et al., 2014), elevated expressions of 2 genes annotated as CSPs, however, were observed in resting cysts of *S. trochoidea*, and one of them, the Unigene85973, was further confirmed with qPCR experiment. It was proposed that CSPs do not function in cold adaptation in dinoflagellates but act as regulators of other cellular processes instead during normal growth (Roy et al., 2014). Our data seemed to support this point. Intriguingly, we also found in resting cysts markedly decreased expressions in 3 genes encoding Hsp70s, a class of pivotal molecular chaperones that are central components in cellular folding catalysts network and cellular homeostasis under both optimal and adverse conditions (Feder and Hofmann, 1999; Deng et al., 2015). Since Hsp70s have been well-known in responding to environmental cues, the significantly declined transcriptions of *Hsp70*s in resting cysts of *S. trochoidea* possibly indicate a reduced need for chaperone functions during resting cyst dormancy.

The down-regulated expressions of 17 candidates encoding for chitinases or GLUs in resting cysts primarily indicate the completion of formation of the chitin-containing and glucan-containing cyst wall (Bogus et al., 2014), i.e., the declined expressions of these DEGs would help to protect the structure of cyst wall, since chitinases are specific enzymes with hydrolytic activity directed toward chitins and GLUs are endohydrolase for β-glucans (Cabib, 1987; Bowles, 1990). However, because chitins and β-glucans are also common components of bacterial or fungal surface structures, the expressions of these enzymes, although lowered, would act as agents against infection of chitin- and β-glucans-containing pathogens. Moreover, the strikingly up-regulated expression of the Unigene75034 encoding chitin deacetylase in resting cysts would certainly enhance the ability of resting cysts to prevent from bacterial or fungal infection, because chitin deacetylases catalyze deacetylation of chitin and the product, chitosan, has been well-known for its antibacterial function (Zhao et al., 2010). Given that β-glucans and chitosans are components of cyst wall (Bogus et al., 2014), the thickened wall itself will act as an anti-pathogen chemical barrier.

### Energy metabolism

Energy production is critical for cells' survival and cellular homeostasis. The glycolytic pathway is one of the main fates for glucose, during which a small amount of energy is captured and generate final product of pyruvate. Pyruvate subsequently can be completely oxidized to CO_2_ for efficient production of ATP via TCA cycle and the electron transport system. Noticeably, the resting cysts elicited markedly higher expressions of the predicted pyruvate kinase (PK) (Unigene103016) and putative phosphofructokinase (PFK) (Unigene50054, Unigene87942), which are the key regulatory enzymes of glycolysis, catalyze the irreversible reactions in the pathway (Plaxton, 1996). Similar observations were previously recorded in the higher plant *Populus*, in which the key enzymes of glycolysis were significantly increasingly expressed in the dormant buds (Ning et al., 2013). Besides, the expressions of 5 DEGs (Unigene107654, a putative FBP; Unigene20189, a predicted PC; Unigene41201, a putative aldolase; Unigene32944, a putative aldolase; Unigene42181, a predicted phosphoglycerate kinase) were up-regulated in resting cysts by 6–11 fold. These results together suggest that the glycolytic pathway and TCA were still active during dormancy of *S. trochoidea* cysts at the conditions applied in this study. In natural sediment where cysts are often buried in an anaerobic milieu, however, the TCA process may not be as active as observed here.

We also noticed the preferential expression of a putative malate synthase (MS) gene (CL1648.Contig2) in resting cysts, which is one of the vital enzymes specific to the glyoxylate pathway (Eastmond and Graham, 2001). The glyoxylate cycle, synthesizing carbohydrates from C2 compounds, is usually considered as a bypass of TCA cycle. It is particularly important under carbon limiting conditions. In higher plants, glyoxylate cycle plays a pivotal role in carbohydrates synthesis from storage lipids during seedling (Eastmond and Graham, 2001). Here the enhanced transcription of MS possibly implies that glyoxylate cycle was actively ongoing in resting cysts of *S. trochoidea* to release energy for maintaining respiration and viability. Mitochondrial β-oxidation is the major pathway by which fatty acids are oxidized to yield energy. Before becoming accessible to acyl-CoA oxidase, the first rate-determining enzyme of the pathway, free fatty acids must be converted into acyl-CoA thioesters, which is catalyzed by LACS (Fulda et al., 2002). In *S. trochoidea* resting cysts, among the 4 components (8 DEGs) involved in fatty acid metabolism, the putative *LACS* was significantly down-regulated, suggesting a possibly declined feeding of fatty acid to β-oxidation. Since LACS activity is also important for activating free fatty acid to acyl-CoA, the declined expression of LACS may also indicate a decreased synthesis of fatty acids. However, 3 other components involved in β-oxidation, enoyl-CoA hydratase (accounting for the second hydration step), acetyl-CoA acyltransferase, and acetyl-CoA C-acetyltransferase (catalyzeing the final thiolytic cleavage) greatly increased their expressions in resting cysts. It appeared that β-oxidation process was still active in order to meet energy demand of resting cysts under the conditions applied.

Resting cysts of dinoflagellates are specialized cells with supposedly a minimal activity of metabolism so as to survive an elongated period of time in dormancy (e.g., as long as 100 years in sediments; Ribeiro et al., 2011), which necessitates the expressions of catabolism-relevant genes low enough to consume energy more economically. Ergo, those down-regulated genes encoding catabolic enzymes might be the ones limiting the rate of oxidation. In our study, we did measure significantly lower oxygen consumption in resting cysts than that in vegetative cells, while, however, the respiration seemed still active but lowered in resting cysts. Consistently, the respiration rate of *Scrippsiella hangoei* “temporary” cysts under darkness was measured to be at almost undetectable level (Rintala et al., 2007), and that in resting cysts of *S. trochoidea* was reported to be ~10% of that in vegetative cells during the initial dormancy period and ~1.5% in quiescent cysts (Binder and Anderson, 1990). This is similarly found in resting stages of another phytoplankter, the pelagophyte *Aureoumbra lagunensis*, showed significant reduction in respiration rates when to enter the resting stage and opposite was true for a vegetative stage (Kang et al., 2016). Resting cysts in the natural sediment, however, usually experience a suite of environmental conditions such as lower temperature, limited supply of oxygen, and darkness, which are different from the conditions in which our cysts were maintained (i.e., routine culturing conditions unless otherwise specified) prior to sampling for further global transcriptomic analyses. Therefore, the detected catabolic activity in our resting cysts might be considerably higher than that occurs in the field, which guarantees the need for a more comprehensive investigation in the future, such as how the energy metabolism changes with the elongation of dormancy, temperature, oxygen level, and irradiance.

## Conclusions

In this study, we adopted RNA-seq approach to investigate the transcriptional changes occurring at different life cycle stages of dinoflagellates. A broad range of *in silico* analyses and confirmation experiments for the observed expression patterns via qPCR identified a set of genes possibly regulating the alteration of life cycle stages (i.e., cyst formation and maintenance of dormancy), including genes functioning in processes of phytohormones signal transduction, sexual reproduction and encystment, cell cycle, photosynthesis, energy metabolism, and environmental adaptation. Based on full-length cDNA sequences obtained via RACE-PCR, we confirmed the presence of genes involved in ABA synthesis and catabolism, *NCED, ZEP, AAO*, and *ABAH*, in dinoflagellates. Combined results of qPCR and direct measurements of cellular ABA via ELISA and UHPLC-MS/MS strongly suggest that ABA may play a vital role in controlling the encystment and dormancy of dinoflagellate resting cysts. We believe this study provides a significant advancement toward understanding the molecular mechanisms in resting cyst formation and, more generally, life history of dinoflagellate.

Due to the gigantic genome sizes of dinoflagellates, whole genome sequencing for species of this group has been highly challenging. The last decade, however, has seen a rapid growth in transcriptomic studies for this unique group of eukaryotes, which shed light on the genetic bases of many functional traits and ecological properties. However, it has been found that only about 10–27% of genes are regulated transcriptionally in dinoflagellates (Lin, 2011). A reliance on post-transcriptional mechanisms has been proposed to be evolved by dinoflagellates to accommodate the unusual structural features of their nuclear environment. Therefore, much more work awaits whole genome sequencing with the emerging third-generation full-length sequencing and integration of multi-omics data in order to illuminate the molecular mechanisms underlying numerous attributes of dinoflagellates.

## Author contributions

YT designed the research, provided feedback on the experiments and results, and edited the manuscript; YD performed the majority of experiments and wrote the article with contributions of all authors; ZH maintained the algal cultures and prepared the samples; LS performed the ABA measurements; QP provided technical assistance to LS. All authors read and approved the final manuscript.

### Conflict of interest statement

The authors declare that the research was conducted in the absence of any commercial or financial relationships that could be construed as a potential conflict of interest.
