# Supplementary figures and images for: Transcriptomic Analyses of Scrippsiella trochoidea Reveals Processes Regulating Encystment and Dormancy in the Life Cycle of a Dinoflagellate, with a Particular Attention to the Role of Abscisic Acid
Source: Front Microbiol. 2017 Dec 11;8:2450. doi: 10.3389/fmicb.2017.02450 (PMC5732363; doi:10.3389/fmicb.2017.02450)

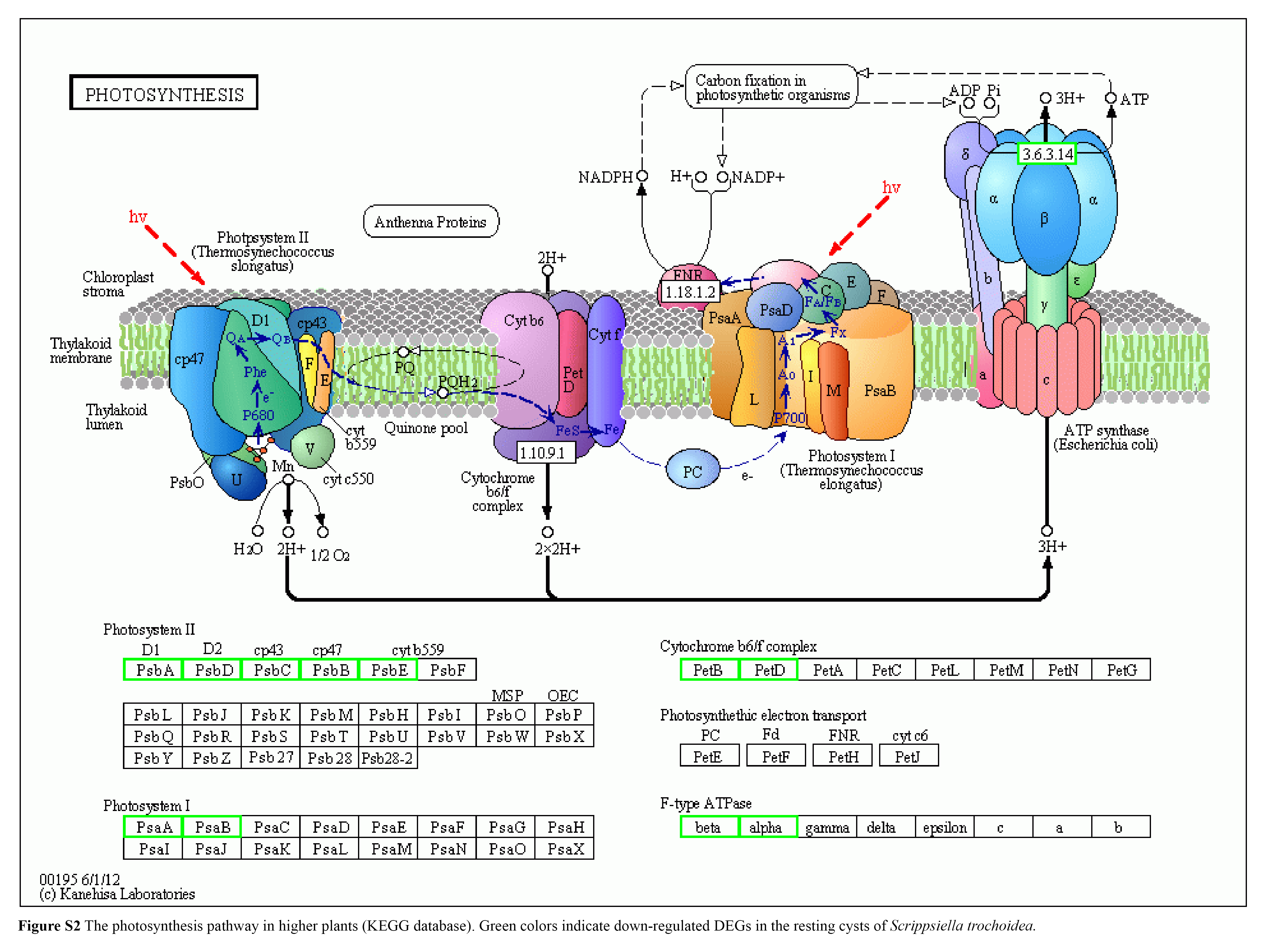

Supplement: Supplementary file 1 [file Image2.tif]
