# Supplementary material for: Transcriptomic Analyses of Scrippsiella trochoidea Reveals Processes Regulating Encystment and Dormancy in the Life Cycle of a Dinoflagellate, with a Particular Attention to the Role of Abscisic Acid
Source: Front Microbiol. 2017 Dec 11;8:2450. doi: 10.3389/fmicb.2017.02450 (PMC5732363; doi:10.3389/fmicb.2017.02450)
Supplement: Supplementary file 5 [file Table4.PDF]

Table S4 Characterizations of *StZEP*, *StNCED*, *StAAO*, and *StABAH*

For the nucleotide sequences

	The length of full-length cDNA sequence (bp)	The length of 5′-UTR (bp)	The length of 3′-UTR (bp)	The length of ORF (bp)	GC content of ORF (%)	Accession number
StZEP	2169	69	312	1788	64.82	KT0337051
StNCED	1700	17	60	1623	61.18	KR148942
StAAO	4254	42	108	4104	60.28	KR148943
StABAH	1692	145	275	1263	53.44	KR148944

For the deduced amino acid sequences

	The number of amino acid residues (aa)	Predicted molecular weight (KDa)	Estimated isoelectric point
StZEP	595	63.26	5.23
StNCED	540	59.42	5.46
StAAO	1367	145.34	6.32
StABAH	420	47.26	6.61
