# Supplementary material for: Transcriptomic Analyses of Scrippsiella trochoidea Reveals Processes Regulating Encystment and Dormancy in the Life Cycle of a Dinoflagellate, with a Particular Attention to the Role of Abscisic Acid
Source: Front Microbiol. 2017 Dec 11;8:2450. doi: 10.3389/fmicb.2017.02450 (PMC5732363; doi:10.3389/fmicb.2017.02450)
Supplement: Supplementary file 13 [file Table12.PDF]

Table S12. List of primers used in the present study

Primer name	Forward primer sequences (5'→3')	Reverse primer sequences (5'→3')	Amplicon length (bp)	Description
**Cloning of genes involved in ABA biosynthesis and catabolism**				
StNCED-P1	GACGGCAAGAGTCTGGCTAC	GCGGACCATGTCGATAATCACT	498	*StNCED* fragment amplification
StNCED-P2	CCACGGGATGGGTCAGAGCCTATCA		830	*StNCED* 3' RACE-PCR
StNCED-P3	CGAAGATGAGGACGGGCGAGTGATT		750	*StNCED* 3' RACE-PCR
StNCED-P4		CCGGGTCGGGCTGGTACTGGAATCC	905	*StNCED* 5' RACE-PCR
StNCED-P5		TTGTCGCCATCCTTCAATGCTCCCG	620	*StNCED* 5' RACE-PCR
StZEP-P1	CGTCGCCGCTTGCTGCTT	AGCACCAAGAAGCCGGCGGC	1512	*StZEP* fragment amplification
StZEP-P2	CGGCCTCAGCTTCGAGAACTTTGGCTACGC		650	*StZEP* 3' RACE-PCR
StZEP-P3	GAACAGGTTCGCGGTGGACTTCGGTGC		514	*StZEP* 3' RACE-PCR
StZEP-P4		GAGGTCTTCTCCAAAACGGTGACCTTG	450	*StZEP* 5' RACE-PCR
StZEP-P5		CAGCAACAGCACCAAGCAGCAAGCGGC	200	*StZEP* 5' RACE-PCR
StAAO-P1	GCTGCGTCCTTCCTCCTT	TCTGTCCGATGTCCACCAT	2990	fragment amplification
StAAO-P2	GTGGCGGCTGCAGAGGTTGAGCTGGA		1200	3' RACE-PCR
StAAO-P3	ACGGGCGAGTGGCGTGTTCTGTTTGC		700	3' RACE-PCR
StAAO-P4		GTCTTCCACCTTGGCCTCGGAAAGCT	832	5' RACE-PCR
StAAO-P5		AGCAGCTCGCTCAAGGAGGAAGGACG	680	5' RACE-PCR

StABAH-P1	AAGGCGACAGTTCAAACC	CATGTAGTGGAACGGGAAG	480	fragment amplification
StABAH-P2	AGGATGTGCATTGGCTACAAGTTTGCGAAG		490	3' RACE-PCR
StABAH-P3	GAGCTCATCATCTGGCTTATGTGCACCTTG		400	3' RACE-PCR
StABAH-P4		CAAGGCGATAGATGTTGTTGACGACAGGCA	1143	5' RACE-PCR
StABAH-P5		TGGATGAAATGGAGCATGGTTTGAACTGTC	933	5' RACE-PCR
**Validation of qPCR reference genes applicable for *S. trochoidea* at different life stages**				
qStGAPDH	GTGGTGCCAAGAAGGTGATC	CAAGAGGCGTTCGAGACAA	118	qPCR for *GAPDH*
qStActin	CACGGCATTGTCACGAAC	CGTAGAGCGACAGAACAGC	214	qPCR for *Actin*
qStCox1	TTGCCATGAGCTGTATTT	ATGACGGATTCCCAAGAT	174	qPCR for *Cox1*
qStTUA	AACACCTTCTTCAGCGAGACA	TTCCCAATGGTATAGTGACCC	188	qPCR for *TUA*
qStTUB	CCAGTGCGGCAACCAGAT	CAGGCTCCAGGTCCATCA	182	qPCR for *TUB*
qStPEPKR	GAATGCCACCGTTGAGTTG	CTCCGCGAGTGAATGTGC	264	qPCR for *PEPKR*
qStRpS4	CCGCTCATCGTAATGCTCC	CGCCTCCTCCTTCACTATCTT	231	qPCR for *Rp-S4*
qSteIF4E	AGTGGGAGGACAAGATGAATG	GATCATGTTCGCTGGCTCA	143	qPCR for *eIF4E*
qStEF-G	CTTCCCGACCTATTGTGG	GAACGGCTCACTGGCATC	169	qPCR for *EF-G*
qStLBP	ACAGACTTGGCTAACTATTGG	TGCTGAGCTGGACACGAT	106	qPCR for *LBP*
qStMDH	GTTGGCTGCCTTGGACCCT	GCATCTGCGCTCGATGTG	112	qPCR for *MDH*
qStSAM	AGATTGCGTTTGATTGCC	TGATTCCCTGATGCGTGT	123	qPCR for *SAM*

qStUBC	GTCTTGACCTACTACGTGGAGC	CGGGCGTTGTACTGATGG	172	qPCR for *UBC*
qStUBQ	AGCGACTACAACATCCAGAAGG	AAGGCGGGCGTAGCACTT	141	qPCR for *UBQ*
qStCYC	CTACGAATGGTGGGAGACG	TCGCAAGTTAGCGGGACT	147	qPCR for *CYC*
**qPCR analysis of genes involved in ABA biosynthesis and catabolism**				
qStNCED	TGGCGACAACTTCACGGCTCAC	GCTTCACGCAATTCAGAACCTC	152	qPCR for *StNCED*
qStZEP	CGCAAGGGTGCCAAAGGT	AGGTTGCCCGTCCAAGTCG	165	qPCR for *StZEP*
qStAAO	TTCCAGGACATTTGGTTCGC	CAGGCGTTTGCTTTGTTGA	193	qPCR for *StAAO*
qStABAH	GAGCCGATGACAGATGAGG	TGTTGACGACAGGCACCAC	259	qPCR for *StABAH*
**qPCR verification of selected RNA-Seq unigenes**				
qHK	GTTGATTACGAAAAGCGGGTTG	CTGTCCAGAAACTGATCGCAGAA	251	qPCR for Unigene31065
qTR	ATCCGAAAAGAAGGAATAGAAACACC	AAATCTAAGTTCAGATTGCCGATAGTG	218	qPCR for Unigene10081
qUnigene53202	TTATACGGGGCTGGGGCACT	CCCGAATAAGTTAAGAAACGACAGAG	191	qPCR for Unigene53202
qCyclin-B	TGGGCTTGGTGAAGTTGGATC	TCTTGAGGCGCTGTGCTGTG	153	qPCR for Unigene51485
qGID1	GCTGCCCCTGCCGTATGT	CCGTGAGGAGCATCTGGGTG	147	qPCR for Unigene78216
qMPK6	ACCCTCCACTTCAATCCGTCC	CCTCTTCAGCCATACCTCCCAC	262	qPCR for Unigene555
qCSP	ACGGGCTGTACGGGACAAA	GGTCTGCTGGGCAACGATTT	222	qPCR for Unigene85973
qCAT	TGTGAAGGAGTTCGCCCACC	CCCCGAGACCAGCAAGCA	274	qPCR for Unigene99398
qRubisco	CGGAGAACAAGGAAATGAGGATT	CAGGAAGGTTGGCGGGAAA	173	qPCR for Unigene20500

qpetB	TGTAACAGCCGTTCCCGAGTC	CGAAGCATTGCGAAATGTATAAGG	183	qPCR for Unigene29956
qPC	TGCACGGCGAGACCTACG	CCACCAGCACATCGACAACG	219	qPCR for Unigene20189
qFBP	ATCTTAGGGGTGGATGGCTCAA	TGTACTTCCCGCCTTTACTGCTC	172	qPCR for Unigene107654
