# Supplementary material for: Transcriptomic Analyses of Scrippsiella trochoidea Reveals Processes Regulating Encystment and Dormancy in the Life Cycle of a Dinoflagellate, with a Particular Attention to the Role of Abscisic Acid
Source: Front Microbiol. 2017 Dec 11;8:2450. doi: 10.3389/fmicb.2017.02450 (PMC5732363; doi:10.3389/fmicb.2017.02450)
Supplement: Supplementary file 14 [file Presentation1.PDF]

Methods S1. Materials and methods

1. Algal samples preparation in the present study

In culture plates, vegetative cells swim in seawater as they have two flagella, while resting cysts (without flagellum) settle at the bottom. Therefore, it is not difficult to physically separate them.

For cDNA library preparation, fresh vegetative cells were harvested from the cultures at Day 5 (counted from the day of inoculation into flasks); mature resting cysts were obtained from the cultures that had been inoculated for about 60 days. They were washed with fresh sterile filtered seawater several times until no vegetative cell or planozygote observed in the samples by checking under an inverted microscope and then were concentrated by centrifugation. The collected vegetative cells and resting cysts were both immediately used for RNA preparation.

For the purpose of screening appropriate reference genes that would be used in the following qPCR experiments, cells at different life stages, including vegetative cells, immature cysts, mature resting cysts, and resting cysts maintained for 1 month (mo.), were isolated from the cultures by micro-pipetting at Day 5, 35, 60, and 90 (the day of inoculation into flasks was recorded as Day 0) (~ 2×10^4 cells for each sample), washed with sterile filtered seawater in Petri dishes, put immediately in liquid nitrogen, and stored at -80°C before RNA extraction.

For qPCR analyses of genes involved in ABA biosynthesis and catabolism, cells were harvested at different life stages as abovementioned and 2 more arrays were conducted: for one, mature resting cysts were kept at the same conditions as culture maintenance in the original plates for 0, 1, 2, 3, 4, 5, and 6 mo., respectively (3 replicates); for the other, mature resting cysts were kept at 4 ± 1°C in darkness for 0, 1, 2, 3, 4, 5, and 6 mo., respectively (3 replicates). Cysts (~ 2×10^4 cells for each sample) were harvested at the abovementioned different time points, swiftly pelleted in 1.5 mL centrifuge tubes, put immediately in liquid nitrogen, and then stored at -80°C before RNA extraction.

Total RNA for the samples prepared above was extracted using RNeasy Plant

Mini Kit (QIAGEN, Germany) and was treated with RNase-Free DNase Set (QIAGEN, Germany) to remove residual genomic DNA. The quantity and quality of total RNA was analyzed with denaturing-formaldehyde agarose gel electrophoresis and NanoDrop^{TM} 1000 spectrophotometer (Thermo Fisher Scientific, USA). The first strand cDNA was synthesized from total RNA with Oligo $(dT)_{18}$ primer and Reverse Transcriptase M-MLV (TaKaRa, Japan). The cDNA mix was incubated at 42°C for 1 h, terminated by heating at 70°C for 15 min, and then stored at -20°C.

For photosynthesis efficiency and respiration rate measurements, fresh vegetative cells were harvested from the cultures at Day 5 (counted from the day of inoculation into flasks); mature resting cysts were isolated by micro-pipetting from the cultures that had been inoculated 50 days prior. Cysts were washed with fresh sterile filtered seawater several times. All samples were prepared in triplicates and vegetative cells fixed with glutaraldehyde was used as negative control in assessment of photosynthetic performance.

2. Library preparation and sequencing

Poly-(A)-containing mRNA were purified from total RNA using oligo-(dT) conjugated magnetic beads (Illumina, USA). The captured mRNA was interrupted into short fragments by adding the fragmentation buffer provided with the Illumina cDNA synthesis kit. With these short fragments as templates, random hexamer primers were used to synthesize the first-strand cDNA. The second-strand cDNA was synthesized using buffer, dNTPs, RNase H, and DNA polymerase I. These double-stranded cDNA fragments underwent process of end repair, addition of a single ‘A’ base and ligation of Illumina adapters (Illumina, USA). Adaptor modified fragments were selected by gel purification and amplified through PCR to create the final mRNA-Seq library. The mRNA-seq libraries were sequenced via the Illumina HiSeq™ 2000 platform that generated about 90 bp paired-end raw reads.

3. Transcriptome data annotation

All the non-redundant unigenes were used for blast search and annotation against the NCBI non-redundant protein sequences (Nr) database, SwissProt database, Gene

Ontology (GO) database, Kyoto Encyclopedia of Genes and Genomes (KEGG) database, and Cluster of Orthologous Groups (COG) database with a 10^{-5} E-value cutoff. The best aligning results were used to determine the sequence direction. Functional annotation of GO terms was performed using Blast2go program (Conesa et al., 2005). WEGO was used to classify GO function (Ye et al., 2006). Unigenes were also compared to the COG database to predict and classify possible functions based on orthologies (Tatusov et al., 2008). Pathway assignments were determined against the KEGG database, which contains graphical representation of biological processes (Kanehisa et al., 2008).

4. Transcriptional profiles of genes involved in ABA biosynthesis and catabolism with qPCR detection

The qPCR was conducted with the SYBR® *Premix Ex Taq*™ (TaKaRa, Tokyo, Japan) on the Eppendorf Mastercycler® ep realplex S (Eppendorf, Germany). The reaction was performed in a volume of 20 μL, which contained 10.0 μL 2×SYBR Premix (TaKaRa, Tokyo, Japan), 1 μL template cDNA, 0.4 μL of each primer (10 μmol/L) (refer to Table S12) and 8.2 μL RNase-free water. The cycling conditions were 95°C for 5 s, followed by 40 cycles of 95°C for 5 s, 50°C for 30 s and 72°C for 30 s. The baseline was set automatically with the software for maintaining consistency. The specificity of each pair of primers was checked with dissociation curves. Relative standard curves for the gene transcripts were generated with serial 10-fold dilutions of cDNA. The qPCR efficiency (*E*) was determined with the slope of a linear regression model (Pfaffl, 2001) and calculated according to the equation: $E = (10^{[-1/slope]} - 1) \times 100$ (Radonic et al., 2004). In order to confirm correct amplification, the qPCR products were migrated on 1% agarose gel and the target bands were purified with agarose gel DNA fragment recovery kit (TaKaRa, Tokyo, Japan), ligated with pMD-19T vector (TaKaRa, Tokyo, Japan), and sequenced (Sangon, Shanghai, China). Each sample was analyzed in 3 replicates and the mean C_T value was calculated. Non-template reactions (NTC) were run as controls.

References

Conesa A, Götz S, García-Gómez JM, et al (2005) Blast2GO: a universal tool for annotation, visualization and analysis in functional genomics research. Bioinformatics 21: 3674-3676

Kanehisa M, Araki M, Goto S, et al (2008) KEGG for linking genomes to life and the environment. Nucleic Acids Res 36: D480-484

Lin S, Cheng S, Song B, et al (2015) The *Symbiodinium kawagutii* genome illuminates dinoflagellate gene expression and coral symbiosis. Science 350: 691-694

Pfaffl M (2001) A new mathematical model for relative quantification in real-time RT-PCR. Nucleic Acids Res 29: 2002-2007

Radonic A, Thulke S, Mackay I, et al (2004) Guideline to reference gene selection for quantitative real-time PCR. Biochem Bioph Res Co 313: 856-862

Tatusov RL, Galperin MY, Natale DA, Koonin EV (2008) The COG database: a tool for genome-scale analysis of protein functions and evolution. Nucleic Acids Res 28: 33-36

Ye J, Fang L, Zheng H, et al (2006) WEGO: a web tool for plotting GO annotations. Nucleic Acids Res 34: W293-297
