# Supplementary material for: Transcriptomic Analyses of Scrippsiella trochoidea Reveals Processes Regulating Encystment and Dormancy in the Life Cycle of a Dinoflagellate, with a Particular Attention to the Role of Abscisic Acid
Source: Front Microbiol. 2017 Dec 11;8:2450. doi: 10.3389/fmicb.2017.02450 (PMC5732363; doi:10.3389/fmicb.2017.02450)
Supplement: Supplementary file 15 [file Presentation2.PDF]

Methods S2. Endogenous ABA quantification

1. Algal samples preparation

For ELISA determinations of endogenous ABA content, vegetative cells (strain IOCAS-St-1) of at exponential and stationary were harvested at Day 5 and 10 (the day of inoculation was recorded as Day 0), respectively. The mature resting cysts were collected as described for cDNA library preparation (Supplemental Methods S1). All samples were prepared in biological triplicates and cells (cysts) were enumerated under an Olympus IX73 inverted microscope, and then stored at -80°C before ABA extraction;

For UHPLC-MS/MS quantification, vegetative cells (strain IOCAS-St-1) at exponential and stationary growth stages were harvested at Day 5 and 10, 15 (the day of inoculation was recorded as Day 0), respectively, for cell density enumeration and ABA quantification. The growth curve was shown in Figure 1. The mature resting cysts were collected as described for cDNA library preparation (Supplemental Methods S1). Mature cysts of another *Scrippsiella trochoidea* strain, BDH-St-1 (LSU and SSU rDNA GenBank accession Nos. KR336540, KR535602), which was established from germination of the sediment collected from Beidaihe River, Hebei Province, China, were also used in UHPLC-MS/MS detection. All samples were prepared in biological triplicates and then stored at -80°C before ABA extraction.

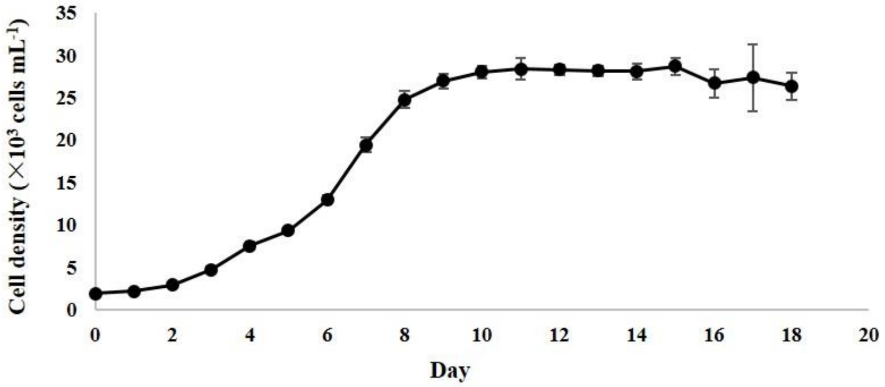

Figure 1. The growth curve of *Scrippsiella trochoidea* (strain IOCAS-St-1) used in UHPLC-MS/MS detection. The day of inoculation was recorded as Day 0.

Values are mean ± standard deviation, *Error Bars* = SD, n=3.

2. Endogenous ABA extraction

The methanol-water extraction solution (80:20, V/V, %) containing 5-di-tert-butyl-4-hydroxytoluol (1 mmol/L) (purity > 99.8%; Solarbio, Beijing, Chin) as an antioxidant was stored at 4 °C prior to use (Lu et al., 2015). The cell or cyst pellets (~ 10^5-10^7 cells) were placed into a 15-mL centrifuge tube with 8 mL extraction solution containing [$^2H_6$]ABA (OlChemIm, Olomouc, Czech Republic) as internal standard (ISTD) and were broken using ultrasonic waves (600 W, 30min) in an ice bath. After extracted at 4 °C overnight, the mixture was centrifuged at 5000 rpm for 5 min at 4 °C and the supernatant was transferred to a 15 mL vial. The residue was re-extracted once with 4 mL of above extraction solution for 4 h and centrifuged to eliminate cell residues. Both supernatants were combined together and dried by nitrogen on a water bath at 35 °C. Samples were re-dissolved in 200 µL of 80% aqueous methanol and stored at -80 °C before analysis.

3. UHPLC-MS/MS Analysis

Samples were injected onto a UHPLC system (Nexera X2 LC-30AD, Shimadzu, Kyoto, Japan) with LC-30AD pumps, SIL-30AC autosampler and a CTO-30A column oven. The UHPLC separation was carried out by an Agilent Poroshell 120 EC-C18 column (4.6 × 150 mm; 2.7 µm) with a flow rate of 0.3 mL/min and column temperature of 40°C. Gradient elution of water /0 .01% formic acid (solvent A) and methanol/0.01% formic acid (solvent B) was used as follows: 0 to 3 min, elution with 30% solvent B; 3 to 10 min, linear gradient of 30% to 70% solvent B; 10 to 15 min, elution with 70% solvent B; 15 to 25 min, elution with 30% solvent B. The injection volume was 10 µL for each analysis.

An AB SCIEX Triple Quad™ 4500 System (ABSciex, Foster City, CA, USA) was used for MS/MS analysis working with Electrospray Ionization (ESI) in negative mode under the multiple reaction monitoring (MRM) conditions. The ionization source parameters were as follows: Temperature (TEM): 350°C; Curtain Gas (CUR): 10 psi; Ion Source Gas 1 (GS 1, nitrogen): 20 psi; Ion Source Gas 2 (GS 2, nitrogen): 20 psi;

Collision Gas (CAD, nitrogen): 8 L/min; IonSpray Voltage (IS): -4000 V. ABA and $[^2H_6]$ABA were monitored at m/z transitions of 263→153 and 269→159, respectively.

Analyst® software version 1.6.2 (AB Sciex) was used for instrument control, data acquisition and data processing. Based on a signal-to-noise ratio of three, the limit of detection (LOD) was 0.1 ng/mL for ABA.

References

Lu Q, Zhang W, Gao J, et al (2015) Simultaneous determination of plant hormones in peach based on dispersive liquid-liquid microextraction coupled with liquid chromatography-ion trap mass spectrometry. J Chromatogr B 992: 8-13
