# Supplementary material for: Transcriptomic Analyses of Scrippsiella trochoidea Reveals Processes Regulating Encystment and Dormancy in the Life Cycle of a Dinoflagellate, with a Particular Attention to the Role of Abscisic Acid
Source: Front Microbiol. 2017 Dec 11;8:2450. doi: 10.3389/fmicb.2017.02450 (PMC5732363; doi:10.3389/fmicb.2017.02450)
Supplement: Supplementary file 16 [file DataSheet1.PDF]

Data S1. Overview of output statistics on *Scrippsiella trochoidea* transcriptome sequencing and the distribution of assembled unigenes.

1. Overview of output statistics on *Scrippsiella trochoidea* transcriptome sequencing

Sample	Total raw reads	Total clean reads	Total Clean nucleotides (nt)	Q20 percentage	N percentage	GC percentage
Cyst-a	66,751,662	51,223,286	4,610,095,740	95.20%	0.01%	61.08%
Cyst-b	57,283,420	51,532,386	4,637,914,740	97.84%	0.00%	60.52%
Cyst-c	58,191,820	52,577,758	4,731,998,220	97.85%	0.00%	61.25%
Vegetative cell-a	59,179,698	53,967,190	4,857,047,100	97.93%	0.00%	60.95%
Vegetative cell-b	60,444,910	54,953,838	4,945,845,420	97.91%	0.00%	61.00%
Vegetative cell-c	56,425,118	51,223,286	4,610,095,740	97.90%	0.00%	60.97%

Q20 percentage = proportion of nucleotides with quality value larger than 20

N percentage = proportion of unknown nucleotides in clean reads

GC percentage = proportion of guanidine and cytosine nucleotides among total nucleotides

2. The distribution of assembled unigenes

Sample	Total number	Total length (nt)	Distinct clusters	Distinct singletons	N50	Mean length (nt)
Cyst-a	197,069	119,339,951	31,096	165,973	1046	606
Cyst-b	202,899	106,609,650	29,501	173,398	868	525
Cyst-c	193,123	121,152,901	31,239	161,884	1084	627
Vegetative cell-a	181,533	120,997,219	30,774	150,759	1174	667
Vegetative cell-b	185,775	117,790,701	30,934	154,841	1115	634
Vegetative cell-c	185,136	113,359,873	29,875	155,261	1067	612
All	166,965	161,746,525	47,677	119,288	1485	969*

The non-redundant unigenes after removal of redundancy were divided into 2 classes based on gene family clustered: one was cluster prefixed with "CL", comprising of several high similar unigenes (more than 70% identities) which may come from same gene or homologous gene; while the other one was singletons prefixed with "CL". Distinct singleton represents unigenes from a single gene. N50 represents the median length of all unigenes.

*The mean length of All was calculated from Unigenes that were obtained by pooling all clean reads from each sample as a whole and a further redundancy removal.

3. Length statistics of assembled unigenes

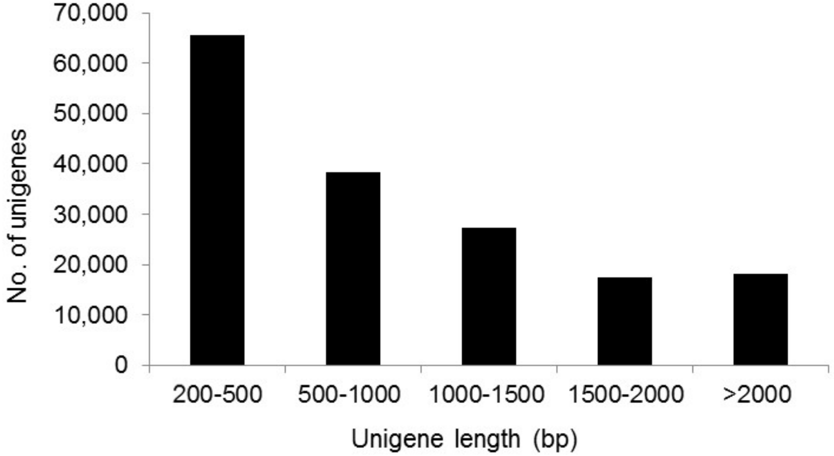

Figure Length dirstribution of non-redundant unigenes. Unigene length ranged from 200 to 12,566 bp. Among them, 65,602 (39.29%) were 200-500 bp in length, 38,437 (23.02%) were 601-1000 bp, 27,316 (16.36%) were 1001-1500 bp, 17,559 (10.52%) were 1501-2000 bp, and 18,051 (10.81%) were longer than 2000 bp in length.
