# Supplementary material for: Transcriptomic Analyses of Scrippsiella trochoidea Reveals Processes Regulating Encystment and Dormancy in the Life Cycle of a Dinoflagellate, with a Particular Attention to the Role of Abscisic Acid
Source: Front Microbiol. 2017 Dec 11;8:2450. doi: 10.3389/fmicb.2017.02450 (PMC5732363; doi:10.3389/fmicb.2017.02450)
Supplement: Supplementary file 17 [file DataSheet2.PDF]

Data S2. Annotation of non-redundant unigenes

The non-redundant unigenes were blasted using the databases Nr, KEGG, GO, COG, and Swissprot databases and 100,788 unigenes, accounting for 60.36% of the total, showed successful blast hits against known sequences in at least one of the above databases. Among them, 97,407 unigenes (58.34%) had significant Nr hits, while 21,627 (12.95%), 70,246 (42.07%), 53,712 (32.17%), and 61,163 (36.63%) unigenes aligned well with sequences in GO, KEGG, COG, and Swissprot databases, respectively (Table 1).

Table 1 Summary of annotation results

Database	Number of annotated unigenes	Percentage of annotated unigenes
Nr	97,407	58.34%
GO	21,627	12.95%
KEGG	70,246	42.07%
COG	53,712	32.17%
Swissprot	61,163	36.63%

A total of 100,788 unigenes (60.36%) are compared well (E-value $<10^{-5}$) with known gene sequences in existing species though Swissprot, Nr, GO, KEGG, and COG databases.

1. GO Annotation

The putative functions of unigenes and their products were identified via searching the annotated genes in GO classifications (Ashburner et al., 2000). A total of 21,627 sequences were assigned to at least one of the 3 main GO categories: biological process, cellular component, and molecular function (Figure 1). These unigenes were further classified into functional subcategories: 20,096 participated in 22 biological processes, including “cellular process” (20.33%), “metabolic process” (17.77%), “single-organism process” (12.42%), and “response to stimulus” (8.15%) comprised the largest proportion; 18,951 unigenes related to 15 cellular components, with the top 4 subcategories being “cell” (25.78%), “cell part” (20.77%), “organelle” (18.75%), and “membrane” (9.63%); 16,328 unigenes involved in 14 molecular functions, with “catalytic activity” (51.99%) being the most abundant, followed by “binding” (34.99%), “structural molecule activity”

(5.86%), and “transporter activity” (5.40%) (Figure 1).

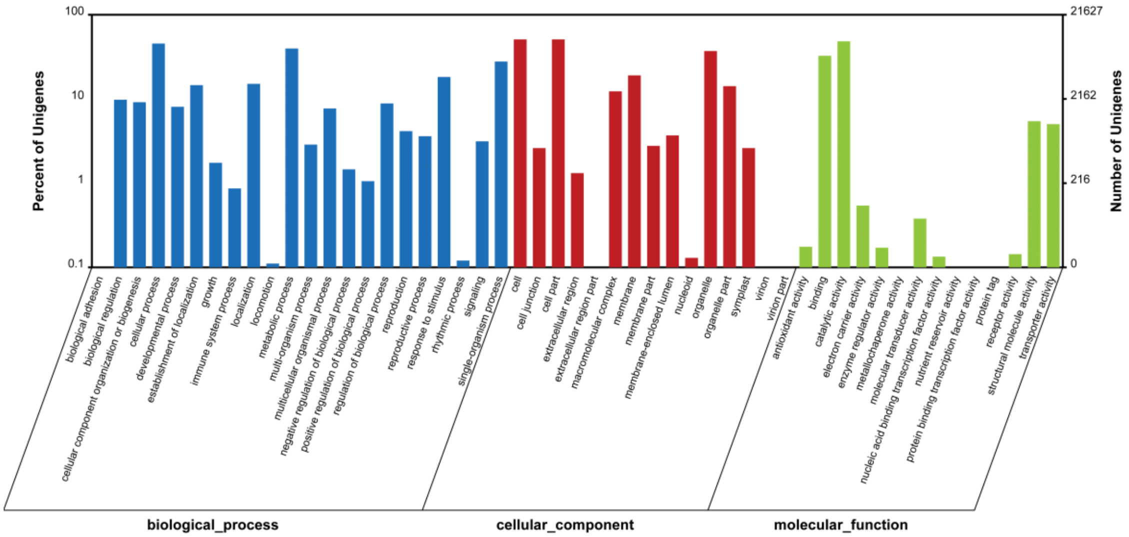

Figure 1 GO annotation of non-redundant unigenes. Good hits are aligned to the GO database and 21,627 transcripts are assigned to at least one GO term. All the unigenes are grouped into 3 major functional categories, namely biological process, cellular component and molecular function. The right y-axis indicates the number of unigenes in a category. The left y-axis presents the percentage of a specific category of unigenes in that main category.

2. COG Annotation

COG is a database where orthologous gene products are classified. Every protein in COG is assumed to be evolved from an ancestor protein, and the whole database is built on coding proteins with complete genome and systematic evolution relationships of bacteria, algae and eukaryotes (Tatusov et al., 2008). COG annotation of our unigenes yielded 53,712 putative proteins in 25 categories (Figure 2). Among these categories, the cluster for “Translation, ribosomal structure and biogenesis” was the largest group (20,221; 37.65%), followed by “General function prediction” (18,242; 33.06%), and “Function unknown” (17,822; 33.18%). Clusters for “Nuclear structure” (2; 0.003%), “RNA processing and modification” (180; 0.335%), and “Chromatin structure and dynamics” (386; 0.719%) were the smallest 3 groups (Figure 2).

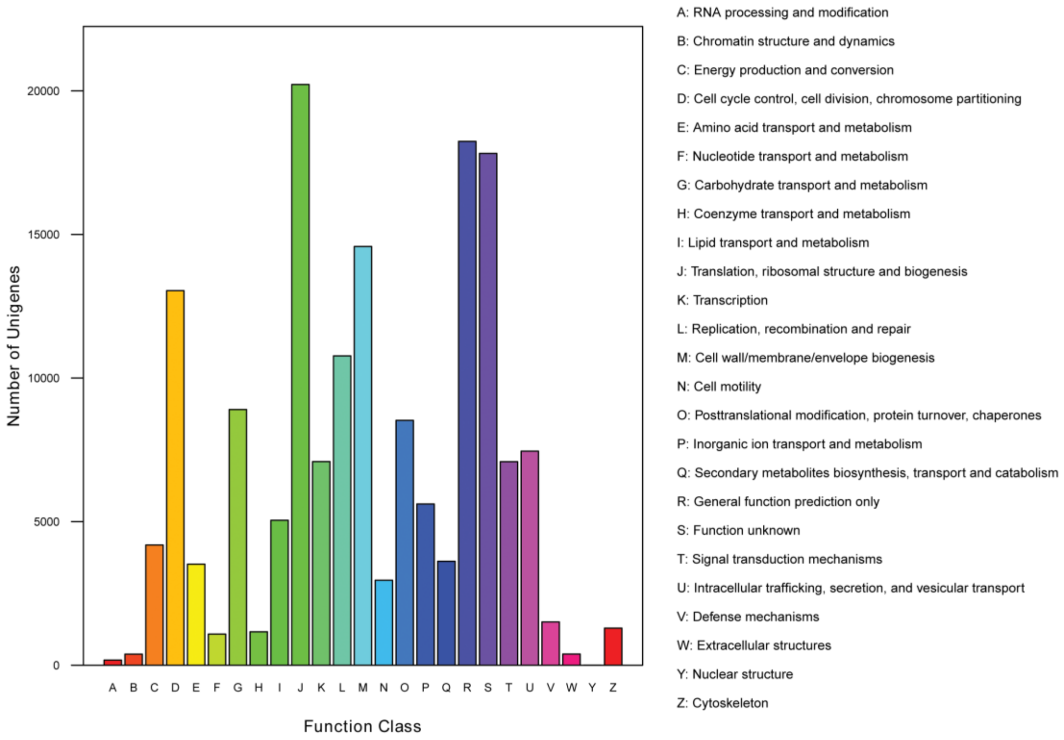

Figure 2 Histogram presentation of clusters of orthologous groups (COG) classification. A set of 53,712 unigenes are assigned to one or more of the 25 classification categories.

3. KEGG Annotation

The KEGG pathway database compiles networks of molecular interactions in cells and their variants specific to particular organisms (Kanehisa and Goto, 2000). To systematically characterize the intracellular metabolic pathways, we mapped the distinct unigenes to the reference pathways in the KEGG database. A total of 70,246 unigenes were assigned to 129 KEGG terms, among which 34,501 members were assigned to “RNA transport”, 30,123 to “mRNA surveillance pathway”, 13,340 to “Metabolic pathways”, 6,072 to “Endocytosis”, 5,693 to “Glycerophospholipid metabolism”, 5,496 to “Ether lipid metabolism”, and others. These KEGG pathways offered a global view of the metabolic pathways at transcriptome level in *S. trochoidea* cells or cysts.

References

Ashburner M, Ball CA, Blake JA, et al (2000) Gene Ontology: tool for the unification of biology. The Gene Ontology Consortium Nat Genet 25: 25-29

Kanehisa M, Goto S (2000) KEGG: Kyoto Encyclopedia of Genes and Genomes. Nucleic Acids Res 28: 27-30

Tatusov RL, Galperin MY, Natale DA, Koonin EV (2008) The COG database: a tool for genome-scale analysis of protein functions and evolution. Nucleic Acids Res 28: 33-36
