# Supplementary material for: Transcriptomic Analyses of Scrippsiella trochoidea Reveals Processes Regulating Encystment and Dormancy in the Life Cycle of a Dinoflagellate, with a Particular Attention to the Role of Abscisic Acid
Source: Front Microbiol. 2017 Dec 11;8:2450. doi: 10.3389/fmicb.2017.02450 (PMC5732363; doi:10.3389/fmicb.2017.02450)
Supplement: Supplementary file 18 [file DataSheet3.PDF]

Data S3. Validation of qPCR reference genes applicable for *Scrippsiella trochoidea* at different life stages

1. Materials and Methods

The culture of *S. trochoidea* at exponential phase was inoculated into 500 mL flasks containing 300 mL medium to achieve an initial cell density of ~ 1 $\times 10^3$ cells mL^{-1} and then grown under the culturing conditions as routine culture maintenance (n=3). The 15 days-old cultures were transferred into 6-well culture plates (Corning, US; 10 mL in each well) for resting cysts production. The cultures were checked for cyst formation every 2 days. Immature cysts (generally displayed circular shape without red body and spine) and mature resting cysts (egg or oval shaped dark cells with a bright red accumulation body inside and numerous surface spines) were identified under an Olympus IX73 inverted microscope according to Qi et al. (1997) and Tang and Gobler (2012). For the purpose of screening appropriate reference genes, cells at different life stages, including vegetative cells, immature cysts, mature resting cysts, and resting cysts maintained for 1 month (mo.) in the incubator, were isolated from the cultures by micro-pipetting at Day 5, 35, 60, and 90 (the day of inoculation into flasks was recorded as Day 0), washed with sterile filtered seawater in Petri dishes, put immediately in liquid nitrogen, and stored at -80°C before RNA extraction.

Based on the transcriptome sequencing data, 15 candidates, glyceraldehyde-3-phosphate dehydrogenase (*GAPDH*), *α*-tubulin (*TUA*), *β*-tubulin (*TUB*), actin (*ACT*), cytochrome oxidase subunit 1 (*COX1*), S4 ribosomal protein (*Rp-S4*), cyclophilin (*CYC*), luciferin-binding protein (*LBP*), S-adenosyl methionine synthetase (*SAM*), ubiquitin (*UBQ*), ubiquitin conjugating enzyme (*UBC*), malate dehydrogenase (*MDH*), elongation factor G (*EF-G*), phosphoenolpyruvate carboxylase-related kinase (*PEPKR*), and eukaryotic initiation factor 4E (*eIF4E*), were selected to assess whether their expressions are stable enough as reference genes to study *S. trochoidea* at different life stages. The qPCR was conducted with the SYBR® *Premix Ex Taq*™ (TaKaRa, Tokyo, Japan) on the Eppendorf Mastercycler® ep

realplex S (Eppendorf, Germany) (see Supplemental Methods S1and Table S12 for more details).In order to confirm correct amplification, the qPCR products were run on 1% agarose gel and the target bands were purified with agarose gel DNA fragment recovery kit (TaKaRa, Tokyo, Japan), ligated with pMD-19T vector (TaKaRa, Tokyo, Japan), and sequenced (Sangon, Shanghai, China).Gene expression stabilities were estimated with 3 softwares: geNorm (Vandesompele et al., 2002), NormFinder (Andersen et al., 2004), and BestKeeper (Pfaffl *et al.*, 2004).

2. Results

a) GeNorm analysis

According to the expression stability values (*M*) of all candidate genes calculated using geNorm (a low *M* value represented stable gene expression), *LBP* and *MDH* were observed to be the first choice for samples from different life stages, followed by *UBC*, *eIF4E*, *SAM*, and then the others. In contrast, *EF-G* was the most variable (Figure S2-1).

Based on the pair-wise variation (*V*) values calculated by geNorm (Figure S2-2), at least 6 HKGs (*V*6/7= 0.144), *LBP*, *MDH*, *UBC*, *eIF4E*, *SAM*, *CYC*, were recommended for normalization.

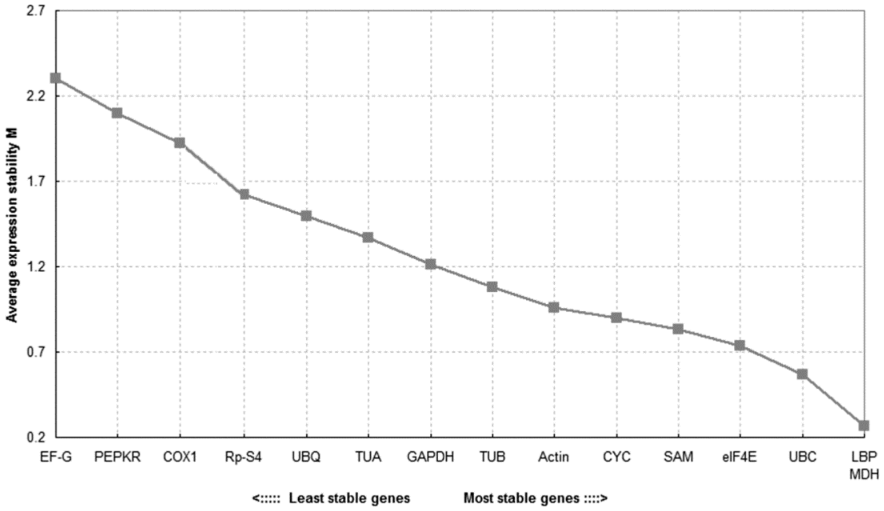

Figure S2-1 Expression stability and ranking of 15 candidate reference genes as calculated by geNorm program. Mean expression stability (*M*) following stepwise exclusion of the least stable gene from the test samples. A lower *M* value indicates more stable expression pattern. The least stable genes are on the left, and the most stable on the right

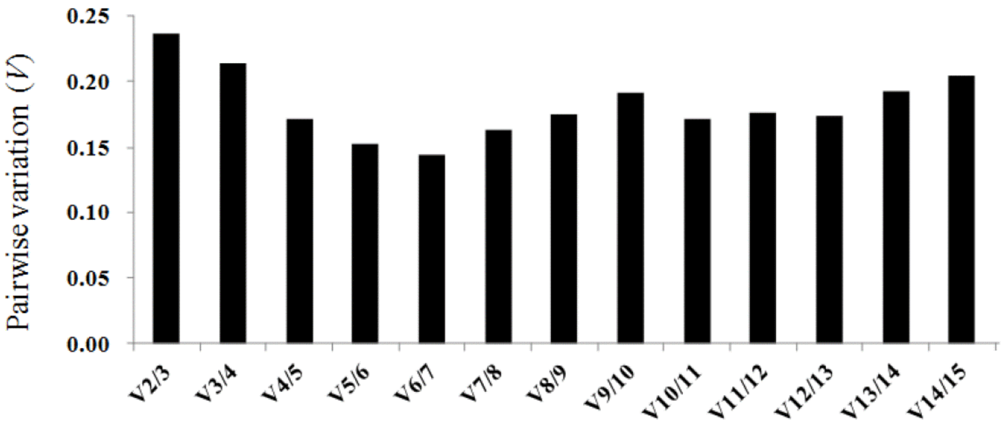

Figure S2-2 Determination of the optimal number of reference genes for normalization using geNorm software. The pairwise variation ($V_{n/n+1}$) was analyzed between the normalization factor NF_n and N_{Fn+1} to determine the optimal number of reference genes required for accurate normalization.

b) NormFinder

Consistent to that observed through geNorm program, *eIF4E*, *MDH*, *SAM*, *CYC*, *LBP*, *UBC* were recommended for normalization purpose (Figure S2-3).

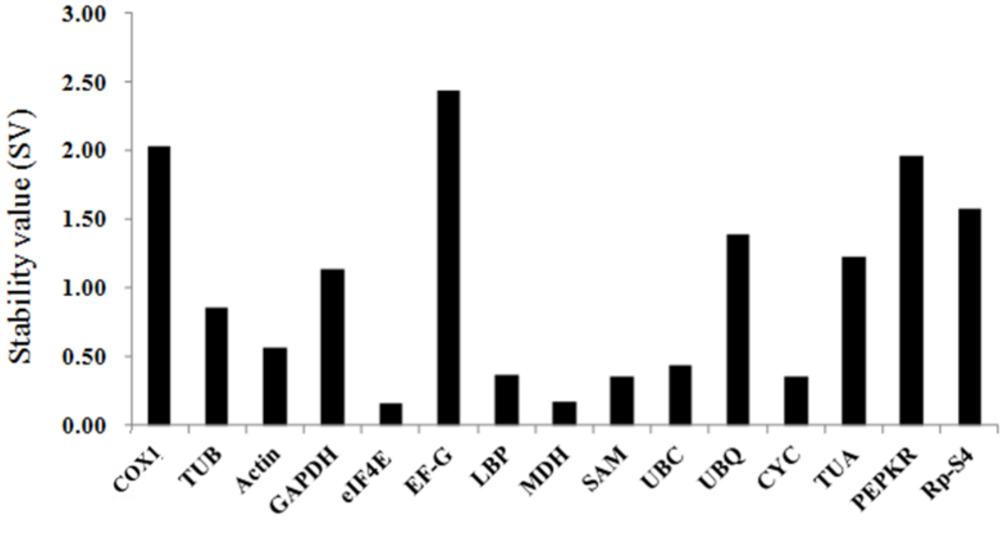

Figure S2-3 NormFinder ranking of candidate genes based on their stability value (SV). Lower SV indicates more stable expression.

c) BestKeeper

In BestKeeper analysis, candidate genes with SD < 1 are generally considered as

stable in expression. Then the program calculated r value between each gene and the BestKeeper index, which was the geometric mean between all stable candidate genes. A higher r value represents a more reliable gene. Figure S2-4 showed the BestKeeper ranking of candidate genes with SD < 1 based on their r values. Consistent to results yielded by geNorm software, *MDH* and *LBP* ranked as highly stable for tested samples, followed by *UBC*, *TUA* and *Rp-S4*.

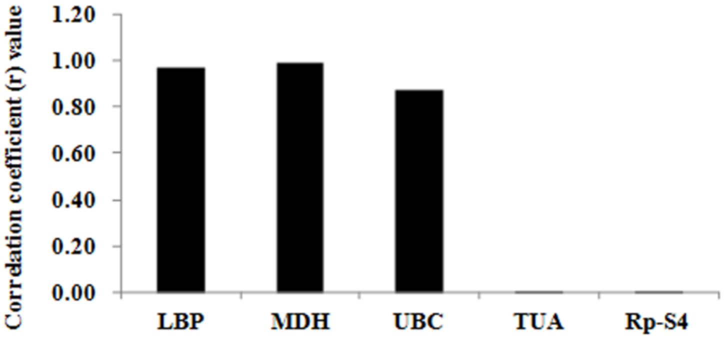

Figure S2-4 BestKeeper ranking of candidate reference genes with standard deviation (SD) < 1 based on their correlation coefficient (r) value.

3. Conclusion

Since calculations of the 3 softwares were principally based on different algorithms, it was not surprising to generate different evaluations for the same gene (de Almeida et al., 2010; Demidenko et al., 2011). Therefore, a comprehensive consideration was taken to identify the best reference genes. In this study, based on the gene expression stabilities, *MDH*, *LBP*, *UBC*, were selected as the reference genes in subsequent qPCR analyses.

References

Andersen CL, Jensen JL, Ørntoft TF (2004) Normalization of real-time quantitative reverse transcription-PCR data: a model-based variance estimation approach to identify genes suited for normalization, applied to bladder and colon cancer data sets. Cancer Res 64: 5245-5250

de Almeida MR, Ruedell CM, Ricachenevsky FK, et al (2010) Reference gene selection for

quantitative reverse transcription-polymerase chain reaction normalization during in vitro adventitious rooting in Eucalyptus globulus Labill. BMC Mol Biol 11: 73

Demidenko NV, Logacheva MD, Penin AA (2011) Selection and validation of reference genes for quantitative real-time PCR in buckwheat (*Fagopyrum esculentum*) based on transcriptome sequence data. PLoS ONE 6: e19434

Pfaffl MW, Tichopad A, Prgomet C, Neuvians TP (2004) Determination of stable housekeeping genes, differentially regulated target genes and sample integrity: BestKeeper-Excel-based tool using pair-wise correlations. Biotechnol Lett 26: 509-515

Qi YZ, Zheng L, Wang R (1997) The life cycle of *Scrippsiella trochoidea* and its physiol-ecological control. Oceanologia et Limnologia Sinica 28: 588-593

Radonic A, Thulke S, Mackay I, et al (2004) Guideline to reference gene selection for quantitative real-time PCR. Biochem Bioph Res Co 313: 856-862

Tang YZ, Gobler CJ (2012) Lethal effects of Northwest Atlantic Ocean isolates of the dinoflagellate, *Scrippsiella trochoidea*, on Eastern oyster (*Crassostrea virginica*) and Northern quahog (*Mercenaria mercenaria*) larvae. Mar Biol 159: 199-210

Vandesompele J, De Preter K, Pattyn F, et al (2002) Accurate normalization of real-time quantitative RT-PCR data by geometric averaging of multiple internal control genes. Genome Biol 3: research0034
