# Supplementary material for: Transcriptomic Analyses of Scrippsiella trochoidea Reveals Processes Regulating Encystment and Dormancy in the Life Cycle of a Dinoflagellate, with a Particular Attention to the Role of Abscisic Acid
Source: Front Microbiol. 2017 Dec 11;8:2450. doi: 10.3389/fmicb.2017.02450 (PMC5732363; doi:10.3389/fmicb.2017.02450)
Supplement: Supplementary file 19 [file DataSheet4.PDF]

Data S4. The relative transcript levels of genes related to ABA biosynthesis and catabolism in the cells at different stages of life cycle (*LBP* and *UBC* used as reference genes).

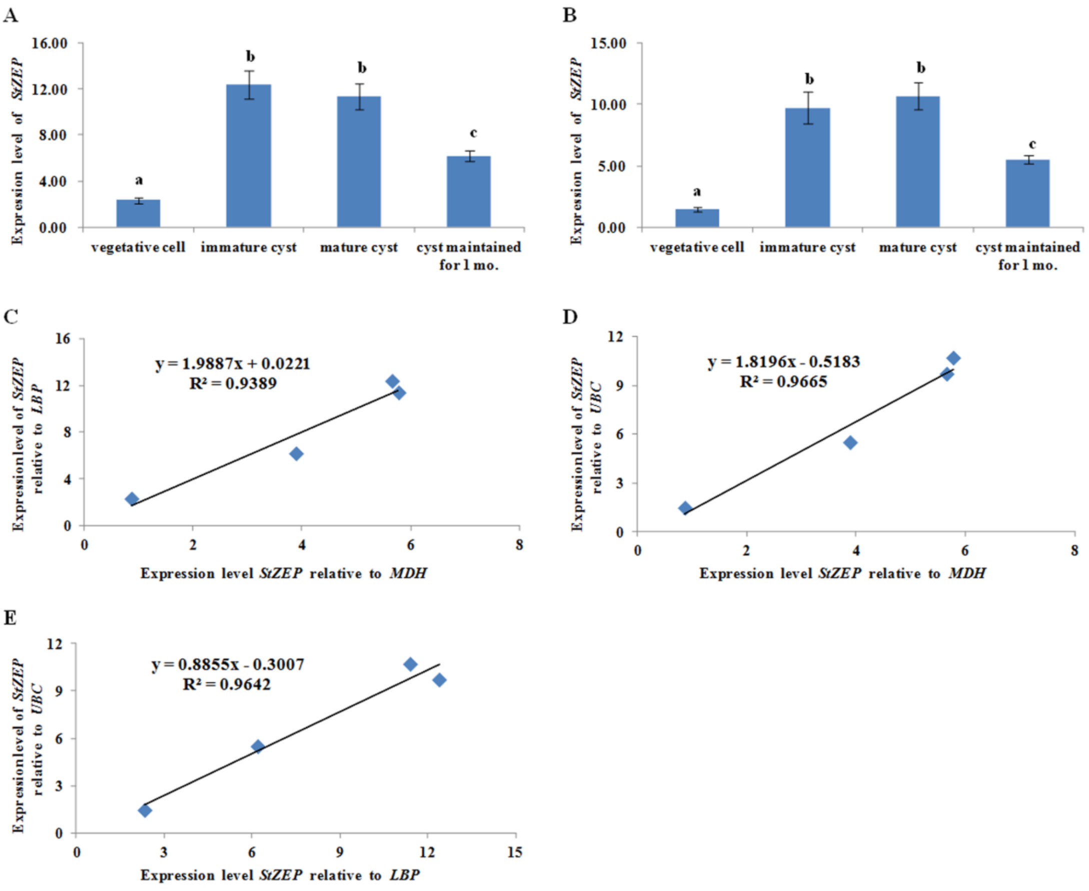

Figure S4-1 *StZEP* transcript levels relative to (A) *LBP* (B) *UBC* in the cells at different stages of life cycle. Significant differences in abundance are indicated with different letters above bars at $p<0.05$; same letter denotes no significant difference. Values are mean ± standard deviation, *Error Bars* = SD, n=3; Correlation of *StZEP* relative expression levels between normalization to (C) *MDH* and *LBP* (D) *MDH* and *UBC* (E) *LBP* and *UBC*.

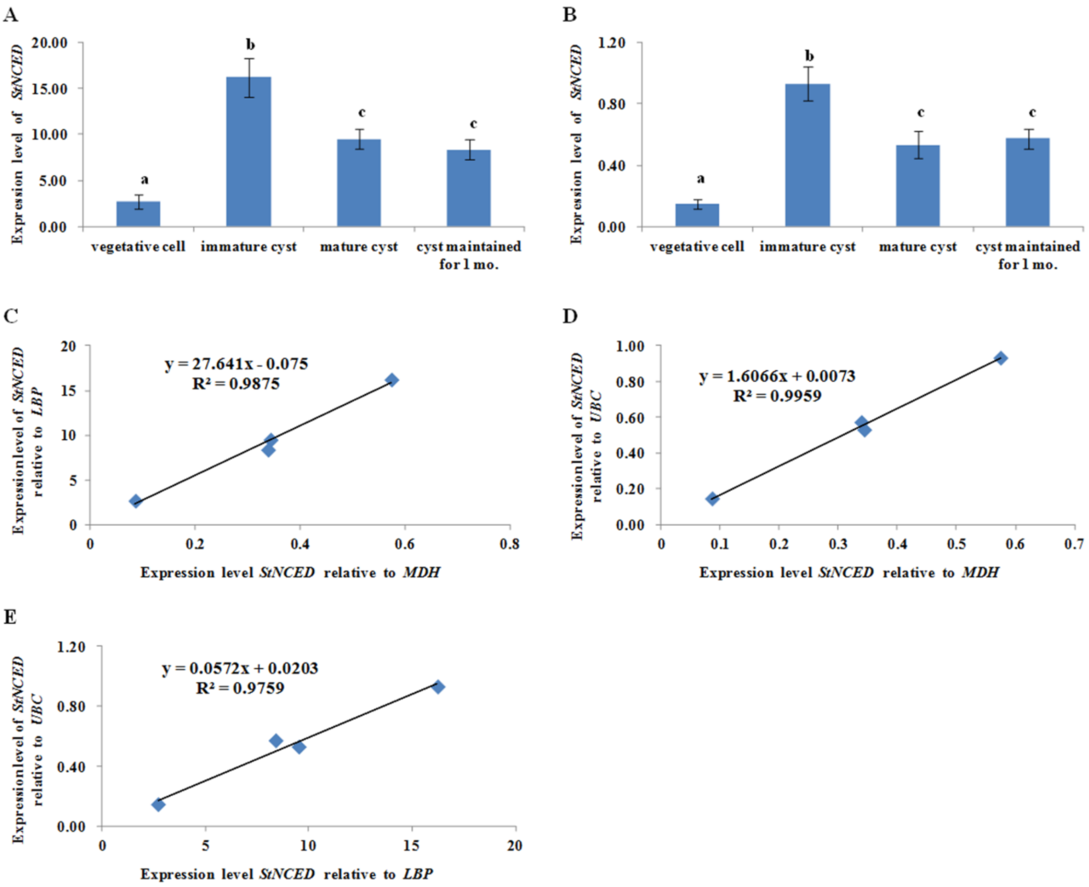

Figure S4-2 *StNCED* transcript levels relative to (A) *LBP* (B) *UBC* in the cells at different stages of life cycle. Significant differences in abundance are indicated with different letters above bars at $p<0.05$; same letter denotes no significant difference. Values are mean ± standard deviation, *Error Bars* = SD, n=3; Correlation of *StNCED* relative expression levels between normalization to (C) *MDH* and *LBP* (D) *MDH* and *UBC* (E) *LBP* and *UBC*.

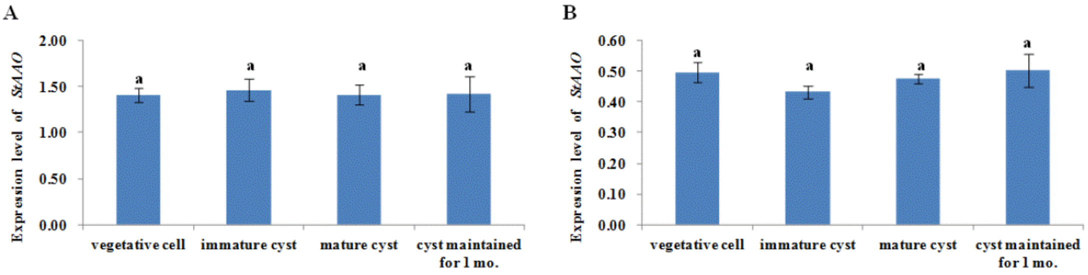

Figure S4-3 *StAAO* transcript levels relative to (A) *LBP* (B) *UBC* in the cells at different stages of life cycle. Same letter above bars denotes no significant difference in abundance at $p<0.05$; Values are mean ± standard deviation, *Error Bars* = SD, n=3.

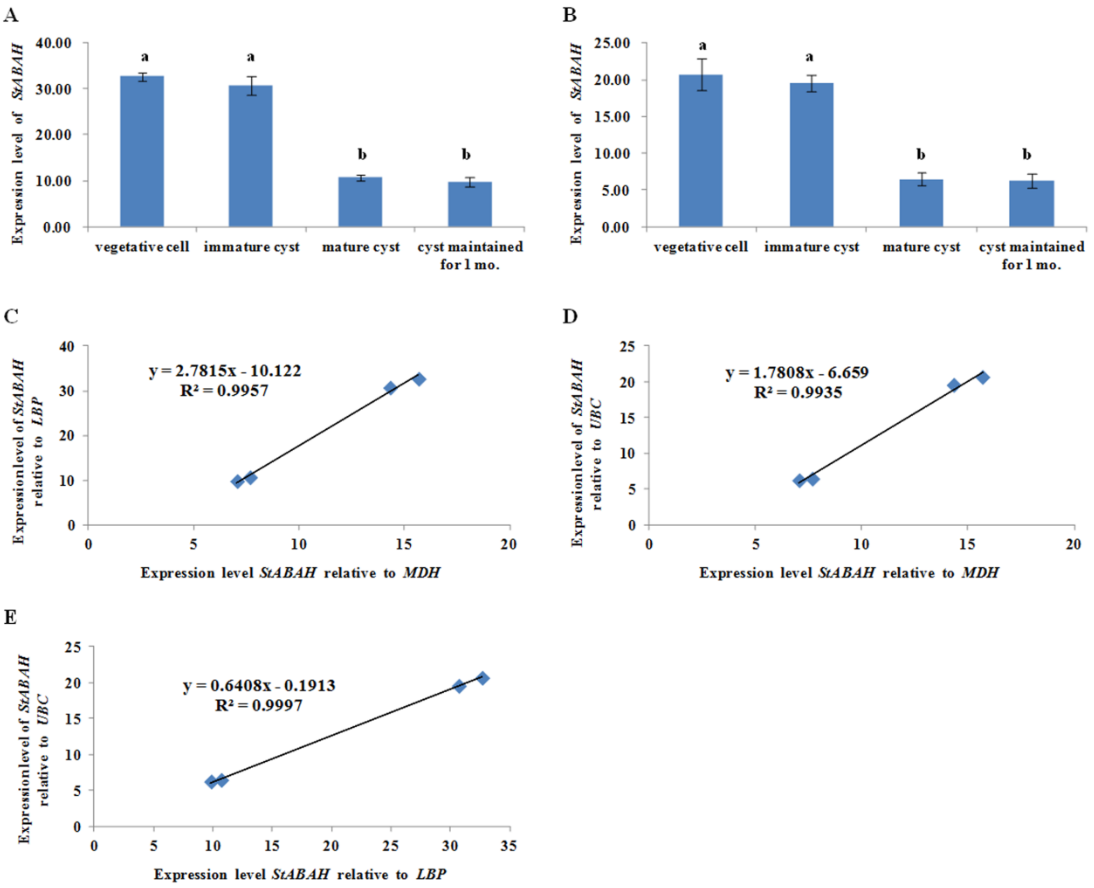

Figure S4-4 *StABAH* transcript levels relative to (A) *LBP* (B) *UBC* in the cells at different stages of life cycle. Significant differences in abundance are indicated with different letters above bars at $p<0.05$; same letter denotes no significant difference. Values are mean ± standard deviation, *Error Bars* = SD, n=3; Correlation of *StABAH* relative expression levels between normalization to (C) *MDH* and *LBP* (D) *MDH* and *UBC* (E) *LBP* and *UBC*.
