# Supplementary material for: Transcriptomic Analyses of Scrippsiella trochoidea Reveals Processes Regulating Encystment and Dormancy in the Life Cycle of a Dinoflagellate, with a Particular Attention to the Role of Abscisic Acid
Source: Front Microbiol. 2017 Dec 11;8:2450. doi: 10.3389/fmicb.2017.02450 (PMC5732363; doi:10.3389/fmicb.2017.02450)
Supplement: Supplementary file 20 [file DataSheet5.PDF]

Data S5. The relative transcript levels of genes associated with ABA biosynthesis and catabolism in the resting cysts maintained at 20 ± 1°C for different periods of time (*LBP* and *UBC* used as reference genes).

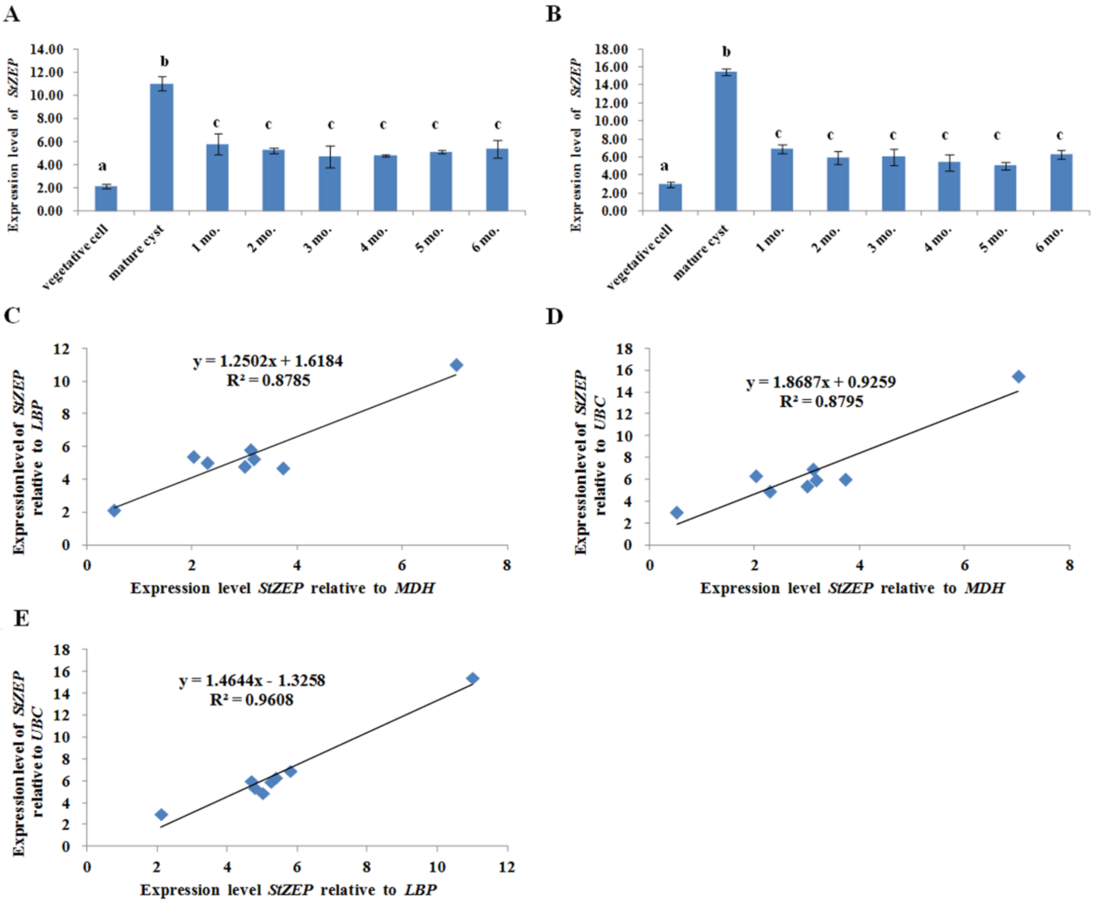

Figure S5-1 *StZEP* transcript levels relative to (A) *LBP* (B) *UBC* in the resting cysts maintained at 20 ± 1°C for different periods of time (0-6 mo.). Significant differences in abundance are indicated with different letters above bars at $p<0.05$; same letter denotes no significant difference. Values are mean ± standard deviation, *Error Bars* = SD, n=3; Correlation of *StZEP* relative expression levels between normalization to (C) *MDH* and *LBP* (D) *MDH* and *UBC* (E) *LBP* and *UBC*.

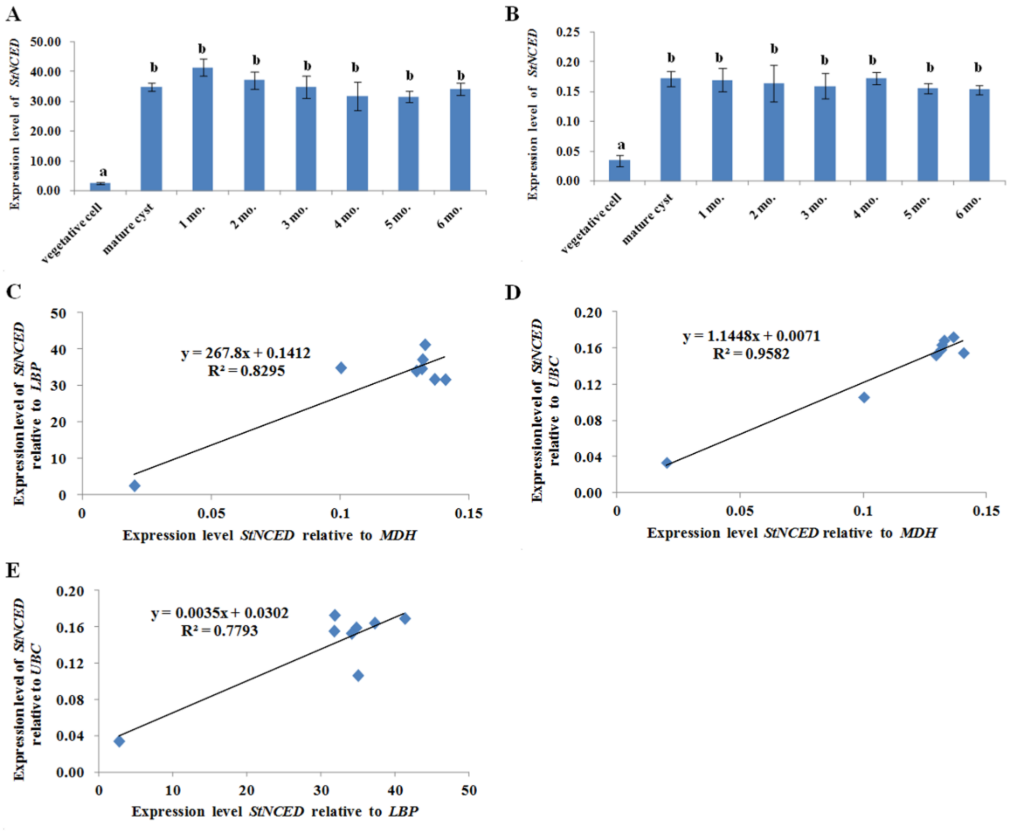

Figure S5-2 *StNCED* transcript levels relative to (A) *LBP* (B) *UBC* in the resting cysts maintained at 20 ± 1°C for different periods of time (0-6 mo.). Significant differences in abundance are indicated with different letters above bars at $p<0.05$; same letter denotes no significant difference. Values are mean ± standard deviation, *Error Bars* = SD, n=3; Correlation of *StNCED* relative expression levels between normalization to (C) *MDH* and *LBP* (D) *MDH* and *UBC* (E) *LBP* and *UBC*.

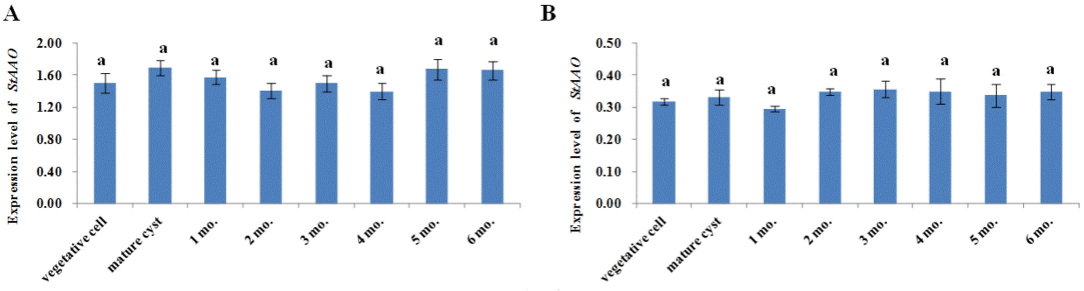

Figure S5-3 *StAAO* transcript levels relative to (A) *LBP* (B) *UBC* in the resting cysts maintained at 20 ± 1°C for different periods of time (0-6 mo.). Same letter above bars denotes no significant difference in abundance at *p*<0.05; Values are mean ± standard deviation, *Error Bars* = SD, n=3.

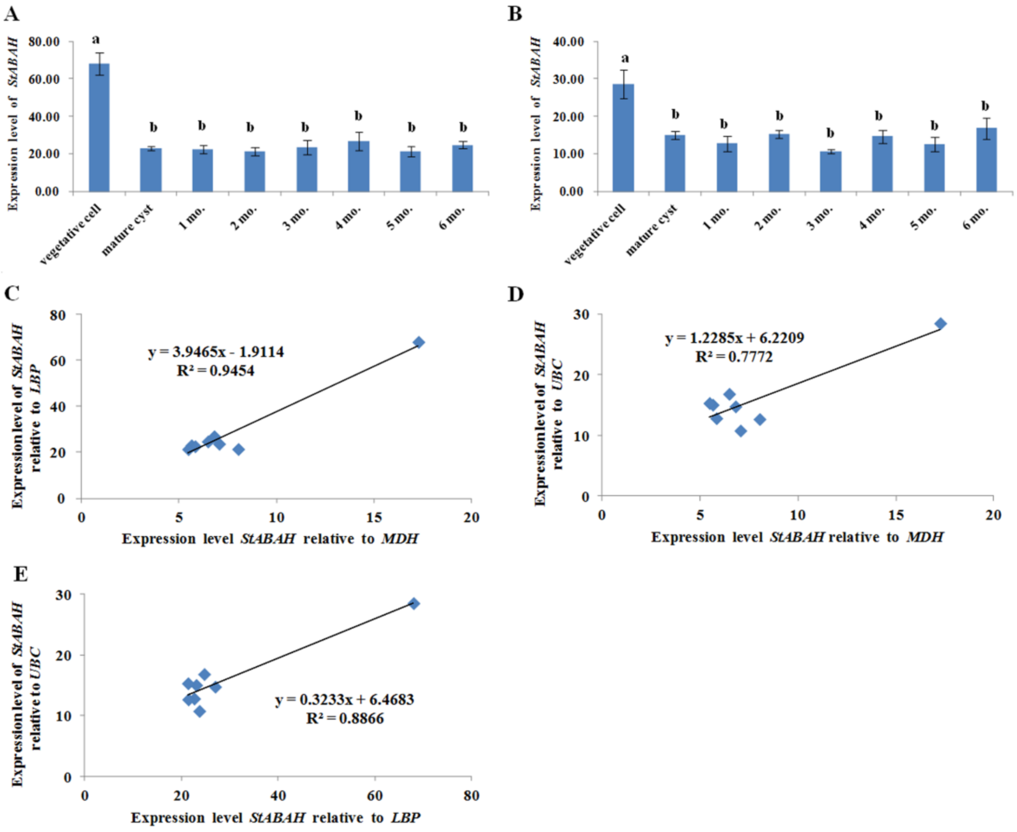

Figure S5-4 *StABAH* transcript levels relative to (A) *LBP* (B) *UBC* in the resting cysts maintained for different periods of time (0-6 mo.). Significant differences in abundance are indicated with different letters above bars at $p<0.05$; same letter denotes no significant difference. Values are mean ± standard deviation, *Error Bars* = SD, n=3; Correlation of *StABAH* relative expression levels between normalization to (C) *MDH* and *LBP* (D) *MDH* and *UBC* (E) *LBP* and *UBC*.
