# Supplementary material for: Transcriptomic Analyses of Scrippsiella trochoidea Reveals Processes Regulating Encystment and Dormancy in the Life Cycle of a Dinoflagellate, with a Particular Attention to the Role of Abscisic Acid
Source: Front Microbiol. 2017 Dec 11;8:2450. doi: 10.3389/fmicb.2017.02450 (PMC5732363; doi:10.3389/fmicb.2017.02450)
Supplement: Supplementary file 22 [file DataSheet7.PDF]

Data S7 Molecular identification of stain IOCAS-St-1.

For molecular identification, the large subunit rDNA gene (LSU) and small subunit rDNA gene (SSU) were amplified from the stain IOCAS-St-1.

Methods

The genomic DNA of vegetative cells was extracted using DNeasy Plant Mini Kit (QIAGEN, Germany) according to the manufacturer's instructions. For the LSU and SSU amplification, the PCR primers were performed as reported in Tang et al. (2012) and Zhang et al. (2012), respectively. PCR was performed in a 25 μL reaction volume, which contained 12.5 μL 2×*TransTaq*™ High Fidelity (HiFi) PCR SuperMix I (TransGen Biotech, Beijing, China), 1 μL genomic DNA of stain IOCAS-St-1, 1 μL of each primer (10 μM) and 9.5 μL RNase free water. The amplification protocol was carried out at 94°C for 5 s, followed by 33 cycles of 95°C for 30 s and 50°C for 1 min, 72°C for 3 min and a final extension at 72°C for 10 min. All the PCR products were purified by agarose gel DNA fragment recovery kit (TaKaRa, ToKyo, Japan) and ligated with pMD19-T vector (TaKaRa, ToKyo, Japan). After being transformed into the competent cells, positive recombinant clones were sequenced (BGI, Beijing, China). The generated sequences were submitted to the NCBI database.

Results

The newly generated partial LSU and SSU sequences were 1432 bp and 1799 bp in length and deposited into GenBank under accession numbers KR336539 and KR535601, respectively. BLAST analysis showed that the KR336539 sequence was 99% identical to the LSU of *Scrippsiella trochoidea* strain CCPO4 (GenBank HQ670228). The KR535601 sequence only deviated from the closest *S. trochoidea* isolate UTEX1017 sequence (GenBank EF492513) by 1%, which was equivalent to 2 base pairs difference. These sequence result confirmed the strain IOCAS-St-1 as *S. trochoidea.*

References

Tang YZ, and Gobler CJ (2012) Lethal effects of Northwest Atlantic Ocean isolates of the dinoflagellate, *Scrippsiella trochoidea*, on Eastern oyster (*Crassostrea virginica*) and Northern quahog (*Mercenaria mercenaria*) larvae. Mar Biol 159: 199-210

Zhang QC, Qiu LM, Yu RC, et al (2012) Emergence of brown tides caused by *Aureococcus anophagefferens* Hargraves et Sieburth in China. Harmful Algae 19: 117-124
