# Supplementary material for: Transcriptomic Analyses of Scrippsiella trochoidea Reveals Processes Regulating Encystment and Dormancy in the Life Cycle of a Dinoflagellate, with a Particular Attention to the Role of Abscisic Acid
Source: Front Microbiol. 2017 Dec 11;8:2450. doi: 10.3389/fmicb.2017.02450 (PMC5732363; doi:10.3389/fmicb.2017.02450)
Supplement: Supplementary file 24 [file Image3.PDF]

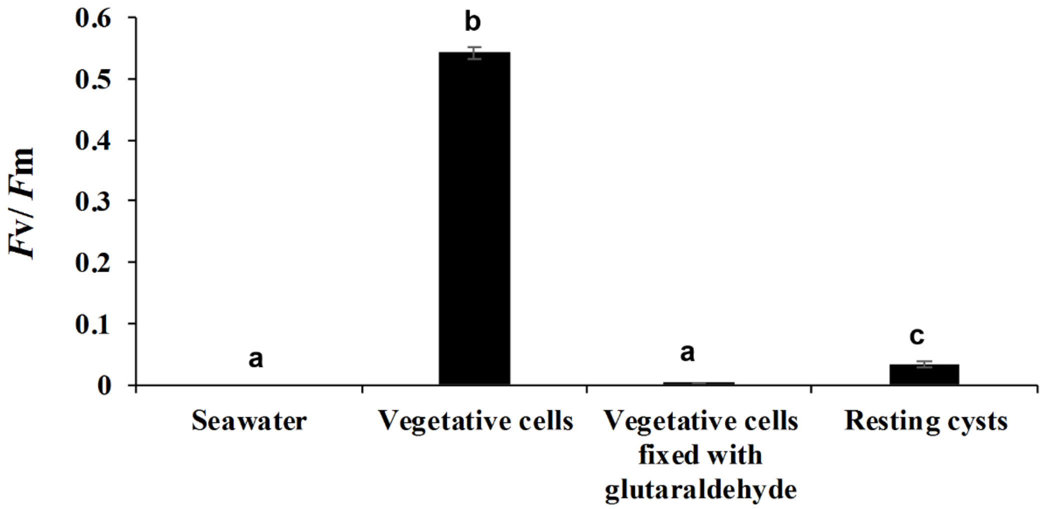

Figure S3. The photosynthetic efficiency (*Fv*/*Fm*) of *Scrippsiella trochoidea*. Significant differences in abundance are indicated with different letters above bars at $p<0.05$; same letter denotes no significant difference. Values are mean ± standard deviation, *Error Bars* = SD, n=3.
