# Supplementary material for: Transcriptomic Analyses of Scrippsiella trochoidea Reveals Processes Regulating Encystment and Dormancy in the Life Cycle of a Dinoflagellate, with a Particular Attention to the Role of Abscisic Acid
Source: Front Microbiol. 2017 Dec 11;8:2450. doi: 10.3389/fmicb.2017.02450 (PMC5732363; doi:10.3389/fmicb.2017.02450)
Supplement: Supplementary file 25 [file Image4.PDF]

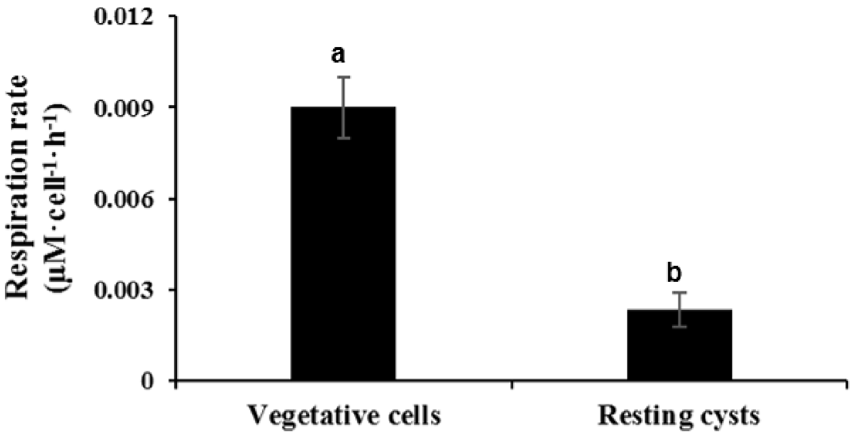

Figure S4. Respiration rates of *Scrippsiella trochoidea* in vegetative cells and resting cysts. Significant differences in abundance are indicated with different letters above bars at p<0.05; Values are mean ± standard deviation, *Error Bars* = SD, n=3.
